# Supplementary material for: Discovery of first-in-class reversible dual small molecule inhibitors against G9a and DNMTs in hematological malignancies
Source: Nat Commun. 2017 May 26;8:15424. doi: 10.1038/ncomms15424 (PMC5458547; doi:10.1038/ncomms15424)
Supplement: Supplementary Information — Supplementary Figures, Supplementary Tables, Supplementary Methods and Supplementary References [file ncomms15424-s1.pdf]

Supplementary Figures

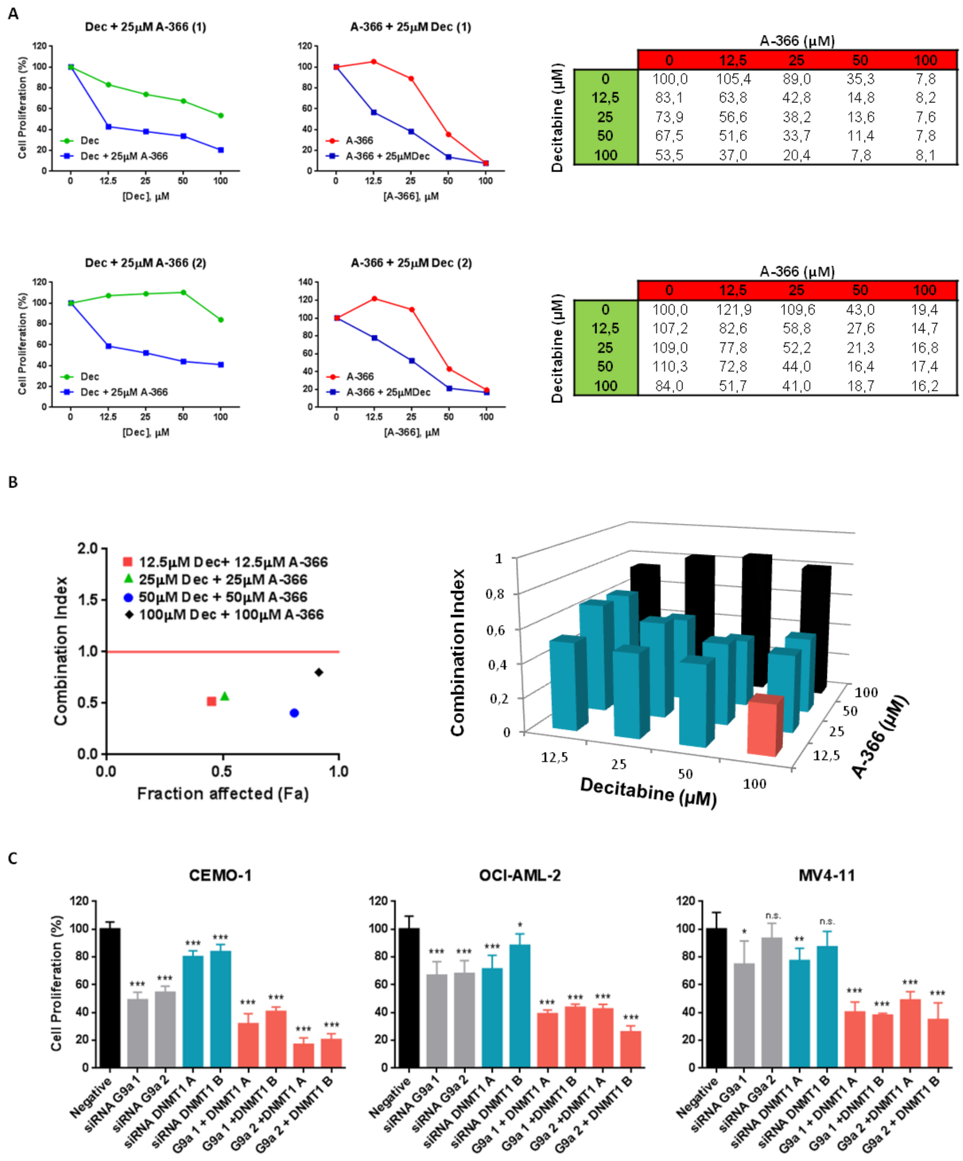

Decitabine (µM) \ A-366 (µM)	0	12,5	25	50	100
0	100,0	105,4	89,0	35,3	7,8
12,5	83,1	63,8	42,8	14,8	8,2
25	73,9	56,6	38,2	13,6	7,6
50	67,5	51,6	33,7	11,4	7,8
100	53,5	37,0	20,4	7,8	8,1

Decitabine (µM) \ A-366 (µM)	0	12,5	25	50	100
0	100,0	121,9	109,6	43,0	19,4
12,5	107,2	82,6	58,8	27,6	14,7
25	109,0	77,8	52,2	21,3	16,8
50	110,3	72,8	44,0	16,4	17,4
100	84,0	51,7	41,0	18,7	16,2

Supplementary Figure 1. Combination study of G9a (A-366) and DNMTs (Decitabine) inhibitors. A) Cell proliferation data of A-366 and Decitabine alone or in combination at 12.5, 25, 50 and 100 µM of each of the drugs in OCI-AML-2 AML cell line. Combination of DNMT1 (Decitabine) and G9a (A-366) inhibitors induced a greater inhibition of cell proliferation than any single compound at any tested concentrations. Results of two biological replicates are shown. B) At any tested concentrations the CI is always lower than 1; according to ranges of CI defined by Chou [1]: Black: moderate synergism (CI between 0.7 and 0.85); Blue: synergism (CI between 0.3 and 0.7); Salmon: strong synergism (CI between 0.1 and 0.3). Combination study set-up was based on GI_{50} values for assayed molecules, A-366 and Decitabine, vs tested cell line, their corresponding GI_{50}s are > 50µM in both cases. Left: synergism level of four different combinations; Right: synergism level of all of the combination tested. A representative example of three different experiments is shown. (C) Cell proliferation analyzes using specific siRNAs against G9a (1 and 2) and DNMT1 (A and B) alone or in combination in CEMO-1, OCI-AML-2 and MV4-11 and cell lines. Asterisk show the P value of a two-tailed Mann-Whitney U test (*=p<0.05; **=p<0.01; ***=p<0.001 and n.s.=no significant). Error bars indicate s.d. from three replicates.

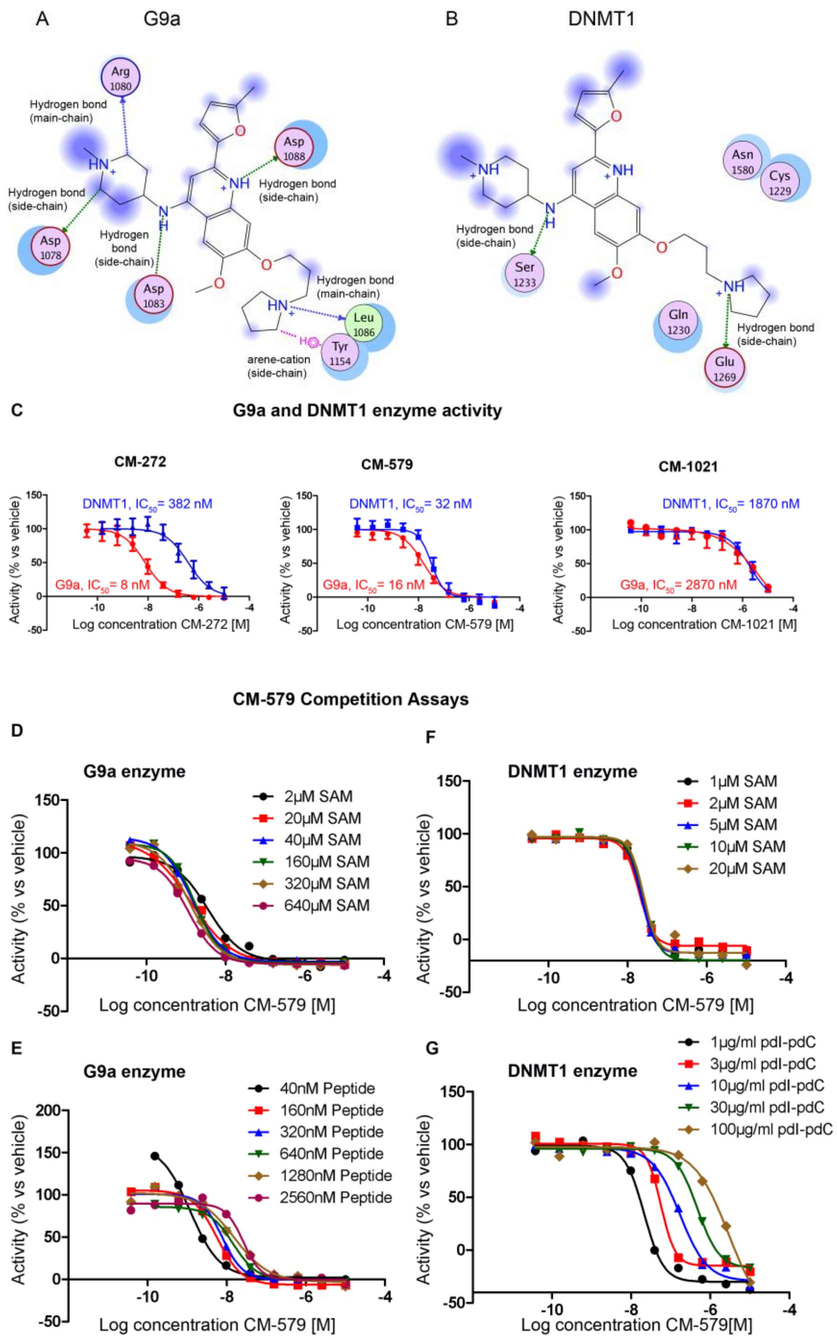

Supplementary Figure 2. CM-272 interaction with G9a and DNMT1. (A-B) According to docking studies, proposed binding modes lead to these key ligand-receptor interactions suggesting the chemical functionalities that should be borne by designed molecules to cover these critical pharmacophoric features. These 2D interaction plots were generated using the MOE program (Chemical Computing Group, http://www.chemcomp.com/), where (A) represents interaction between CM-272 & G9a and (B) CM-272 & DNMT1. (C-G) Small molecules with a dual inhibitor activity against G9a and DNMT1. (C) Dose-response curves of CM-272, CM-579 and CM-1021 inhibition against G9a (red) and DNMT1 (blue). Error bars indicate s.d. from three replicates (D-G) Enzymatic competition assays of CM-579: G9a competition assay with the SAM cofactor (D) and with the histone peptide (PepMe1, E) and DNMT1 competition assay with the SAM cofactor (F) and with the DNA substrate (G).

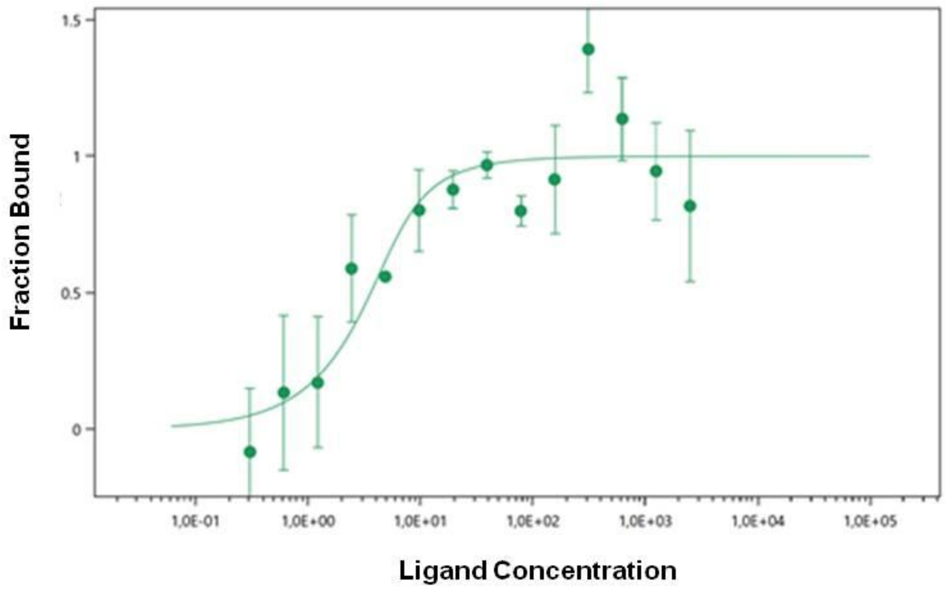

Supplementary Figure 3. Concentrations on the x-axis, corresponding to CM-579, are plotted in nM. This plot represents data from three independent measurements (n=3). A K_d of 1.5 nM was determined for this interaction between DNMT1 and CM-579. Error bars indicate s.d.

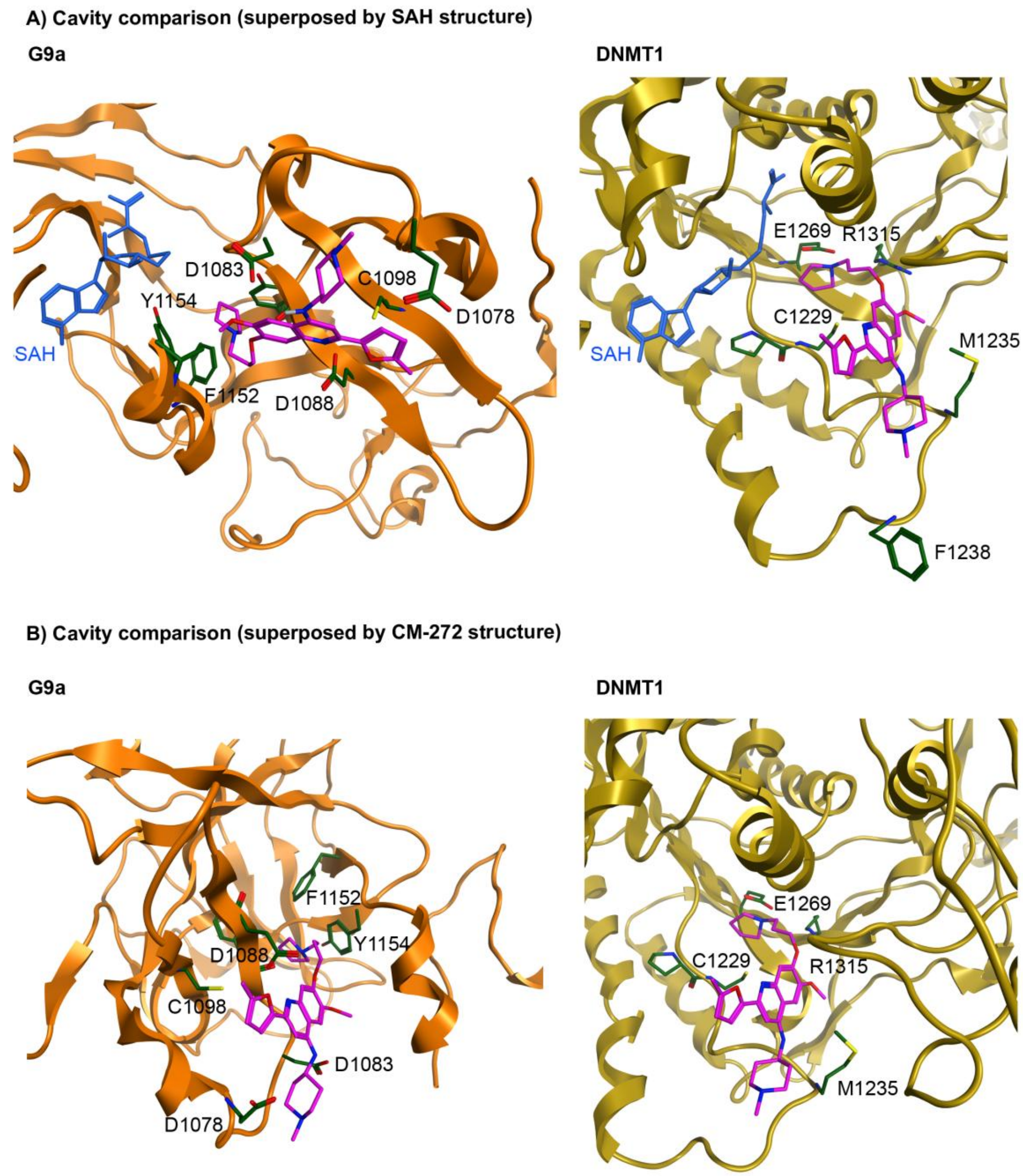

Supplementary Figure 4. Comparison of binding modes of CM-272 into G9a (orange) and DNMT1 (yellow). Given that both proteins are structurally divergent, docked ligand-protein complexes were superposed by the chemical structure of SAH (A, upper figures) and CM-272 (B, lower figures) for the purpose of comparison. SAH and CM-272 are shown in blue and pink sticks, respectively.

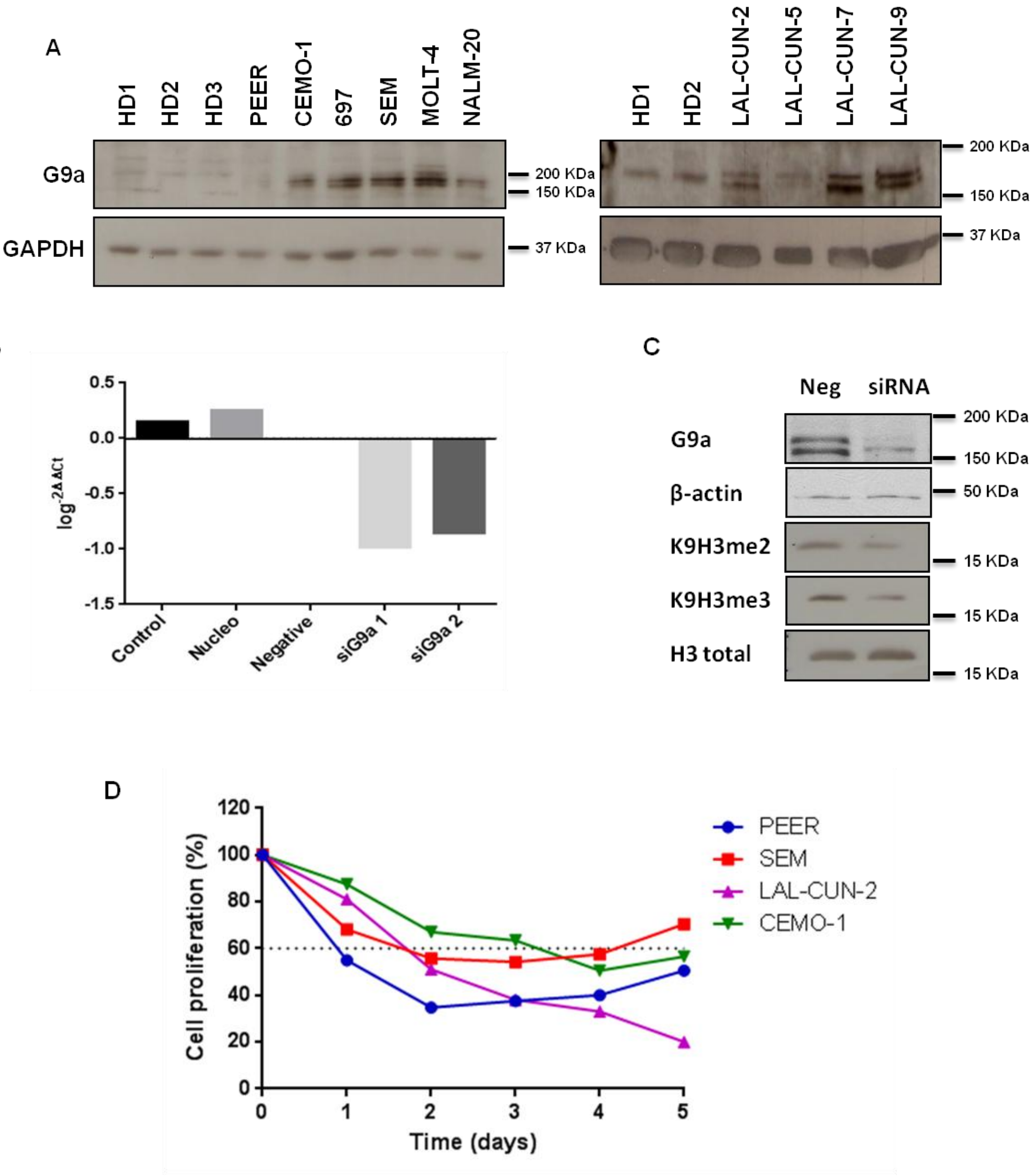

Supplementary Figure 5. (A) G9a levels in several ALL cell lines and primary patient samples in comparison with healthy donors (HD). GAPDH was used as loading control. (B-D) Down-regulation of G9a expression reduces cell proliferation in ALL cell lines. (B) G9a expression was analyzed by Q-RT-PCR 24h after nucleofection with G9a siRNAs. Control: cells without nucleofection; Nucleo: cells nucleofected; Negative: cells nucleofected with the Silencer Select Negative Control 1 (Ambion); siG9a1 and siG9a2: cells nucleofected with G9a siRNAs. (C) Western blot analysis of G9a, H3K9me2 and H3K9me3 48h after nucleofection with G9a siRNA. GAPDH and H3 total were used as loading control. (D) Cell proliferation time course in CEMO-1, PEER, SEM and LAL-CUN-2 ALL derived cell lines after nucleofection with G9a siRNA. A representative example of three different experiments is shown.

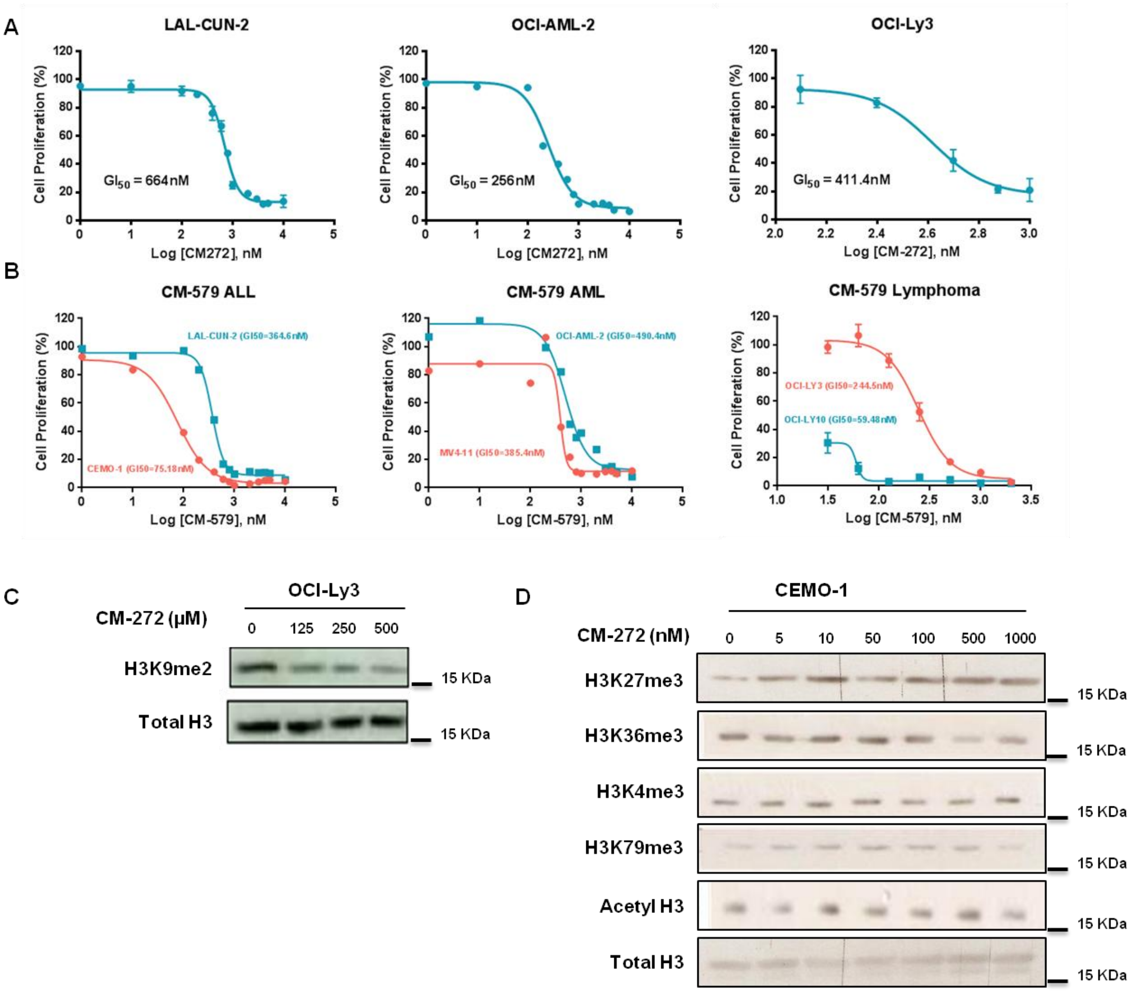

Supplementary Figure 6. CM-272 and CM-579 inhibit cell proliferation in ALL, AML and DLBCL cell lines. (A) GI_{50} values of CM-272 for LAL-CUN-2 ALL cell line, OCI-AML-2 AML cell line and OCI-Ly3 DLBCL cell line. (B) GI_{50} values of CM-579 for CEMO-1 and LAL-CUN-2 cell lines, MV4-11 and OCI-AML-2 cell lines and OCI-Ly3 and OCI-Ly10 lymphoma cell lines. Error bars indicate s.d. from three replicates. (C) H3K9me2 levels after 48 hours of CM-272 treatment with different doses in OCI-Ly3 lymphoma cell line. H3 total was used as loading control. (D) Effect of CM-272 in different histone modifications H3K27me3, H3K36me3, H3K4me3, H3K79me3 and H3 acetylation levels after 48 hours of treatment with different doses of CM-272 in CEMO-1 cells. H3 total was used as loading control.

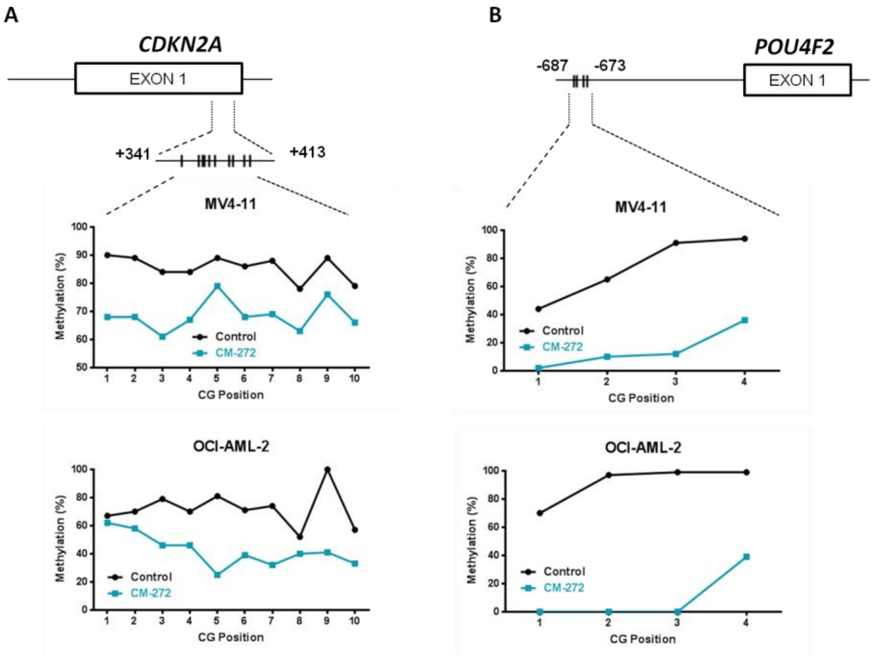

Supplementary Figure 7. DNA methylation analysis. (A-B) CM-272 induces demethylation in promoter region of *CDKN2A* and *POU4F2*. DNA methylation analysis by pyrosequencing of the promoter region of *CDKN2A* (A) and *POU4F2* (B) in AML derived cell lines MV4-11 and OCI-AML-2 and before and after treatment with CM-272. Data shown are a representative experiment of three independent experiments.

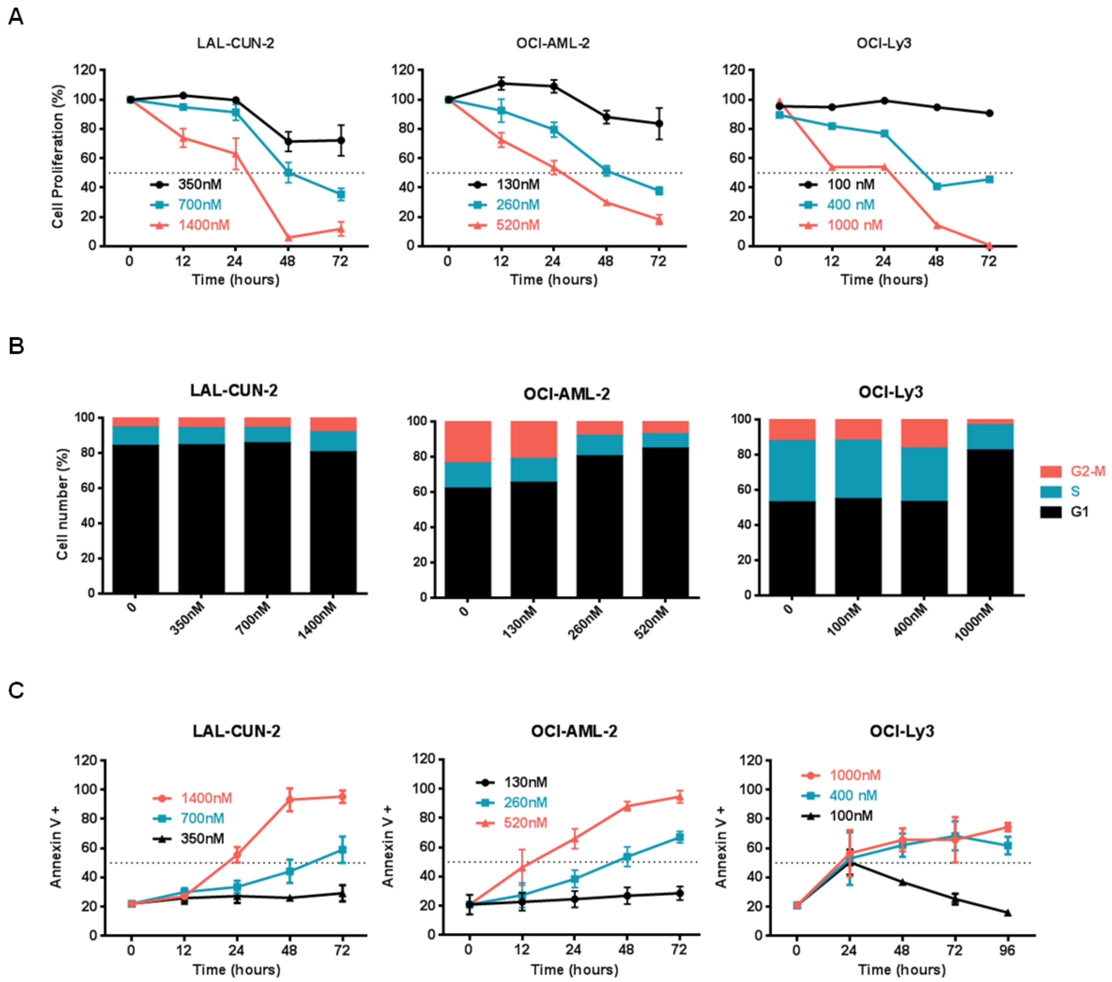

Supplementary Figure 8. CM-272 inhibits cell proliferation and induces apoptosis in ALL, AML and DLBCL cell lines. (A) Cell proliferation time course in LAL-CUN-2, OCI-AML-2 and OCI-Ly3 cell lines treated with different concentrations of CM-272 for 12, 24, 48 and 72 hours. (B) Cell cycle in LAL-CUN-2, OCI-AML-2 and OCI-Ly3 cell lines treated with different concentrations of CM-272 for 24-48 hours. (C) Apoptosis time course in LAL-CUN-2, OCI-AML-2 and OCI-Ly3 cell lines treated with different concentrations of CM-272 for 12, 24, 48 and 72 hours. Error bars indicate s.d. from three replicates.

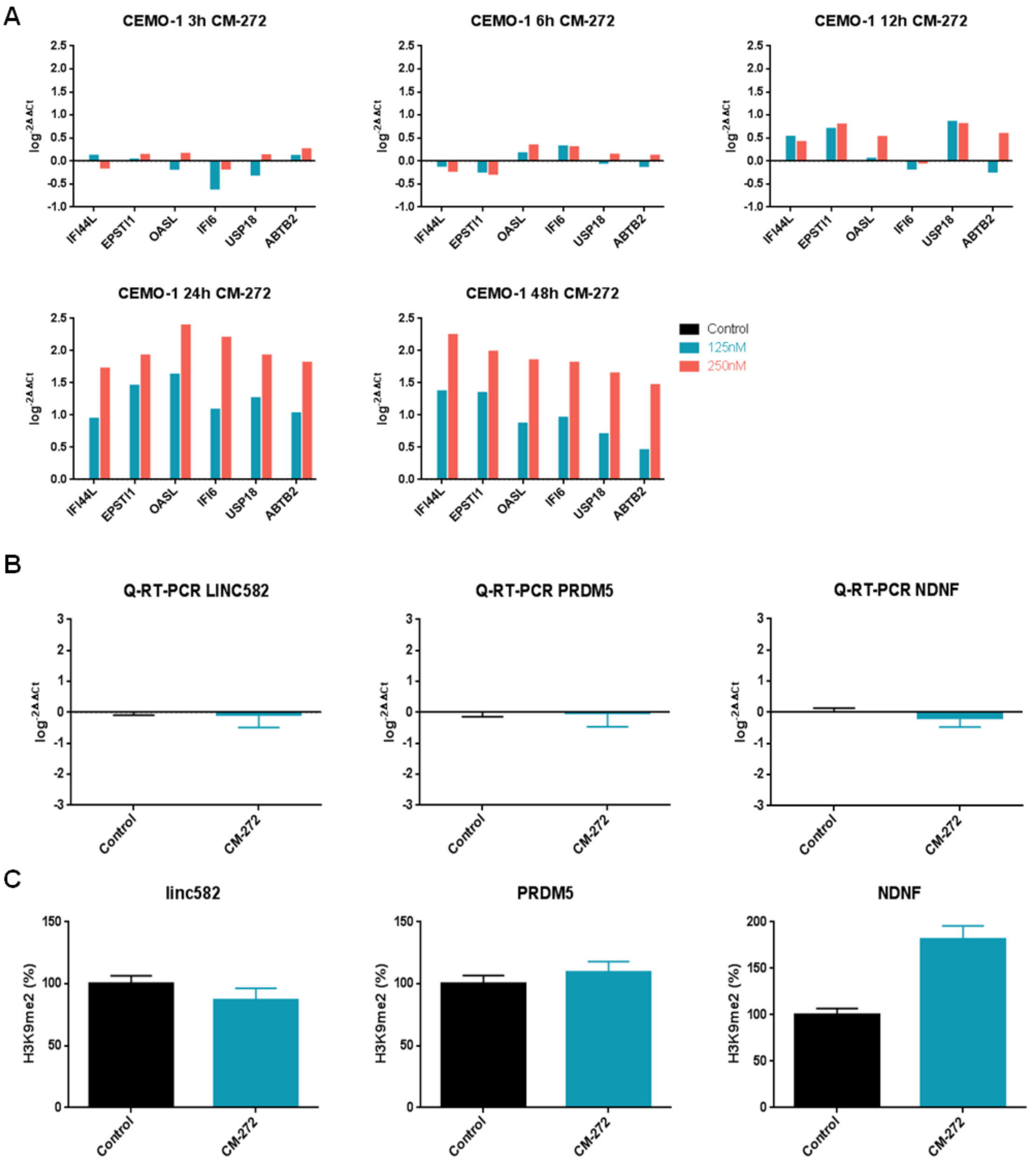

Supplementary Figure 9. mRNA expression and H3K9me2 levels after CM-272 treatment of leukemic cells. (A) CM-272 induces type I interferon response in ALL. RNA-Seq validation by Q-RT-PCR of 6 ISGs genes in CEMO-1 cell line treated with 125 and 250 nM of CM-272 for 3, 6, 12, 24 and 48h. Black: control; Blue: 125 nM CM-272; Salmon: 250 nM CM-272. Data shown are a representative experiment of three independent experiments. (B) Q-RT-PCR validation of *PRDM5*, *NDNF* and *Linc582* in CEMO-1 cell line treated for 48h with 250nM of CM-272. (C) Q-ChIP-PCR analysis of *PRDM5*, *NDNF* and *Linc582* in CEMO-1 cell line treated for 48h with 250nM of CM-272. Error bars indicate s.d. from three replicates.

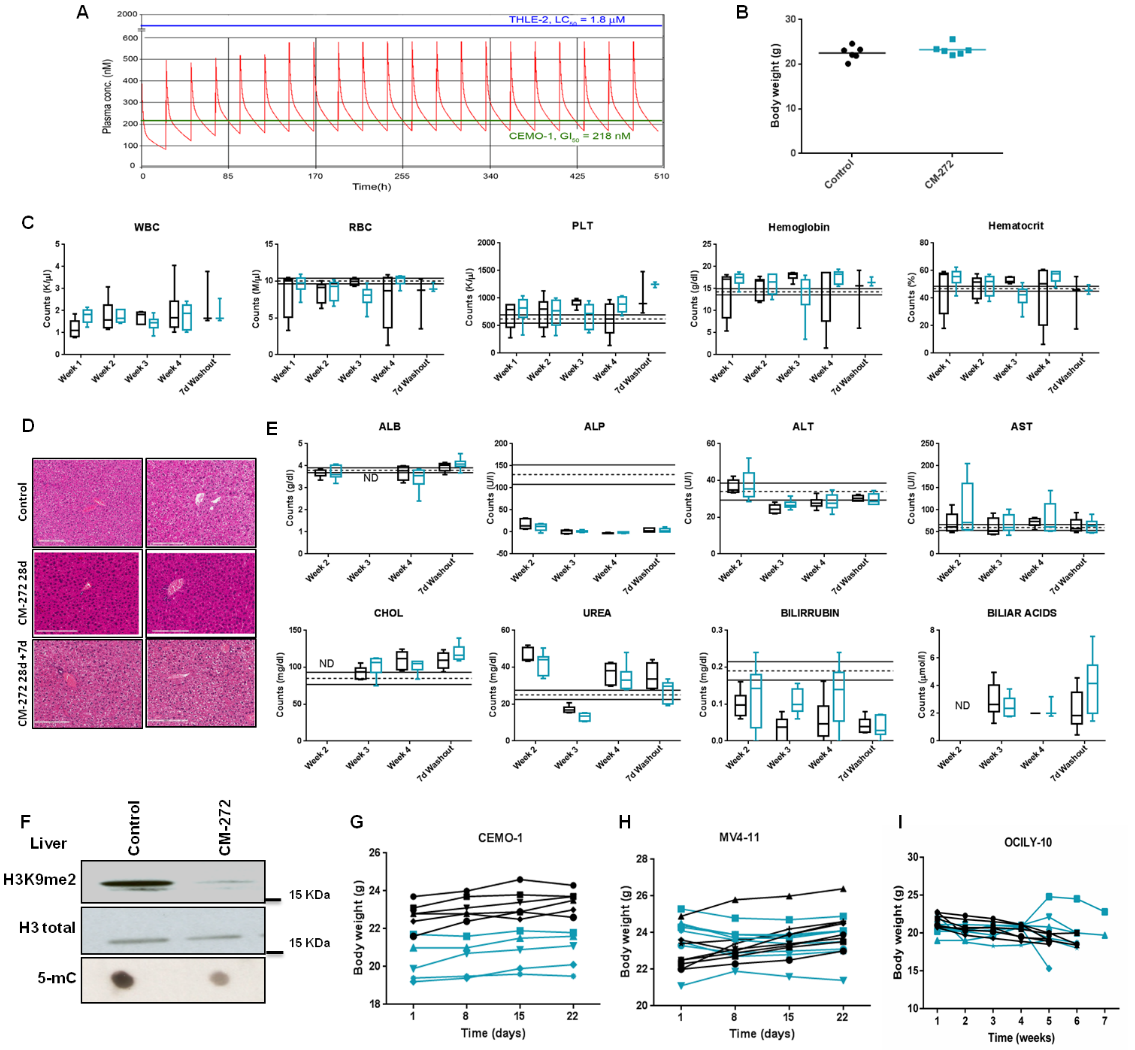

Supplementary Figure 10. CM-272 shows anti-leukemic effects in vivo. (A) Based on the experimental pharmacokinetics results for CM-272, simulated plasma concentration-time profiles for a dosing regimen of 2.5 mg/kg every 24 hours during 3 weeks administered intravenously. GI_{50} of CM-272 for CEMO-1 and LC_{50} tested on THLE-2 cell line are indicated to define the corresponding therapeutic window for CM-272 vs this ALL cell line. (B) Mean ± SEM body weight of healthy mice after 28 days of treatment with CM-272 (Control group with saline solution n=6; Treated group with CM-272 n=6). (C) Hematological parameters in healthy mice treated with vehicle, 2.5 mg/kg daily for 4 weeks (n=6) or 2.5 mg/kg of CM-272 daily for 4 weeks (n=6) followed by a 7 days washout period. WBC: white blood cells; RBC: red blood cells; PLT: platelet count. Black: control, Blue: CM-272 treatment. (D) Hematoxylin and eosin staining for liver tissue in healthy mice treated with vehicle, 2.5 mg/kg daily for 4 weeks or 2.5mg/kg of CM-272 daily for 4 weeks followed by a 7 days washout period. (E) Hepatic parameters in healthy mice treated with vehicle, 2.5 mg/kg daily for 4 weeks (n=6) or 2.5 mg/kg of CM-272 daily for 4 weeks (n=6) followed by a 7 days washout period. ALB: albumin; ALP: alkaline phosphatase; AST: aspartate transaminase; ALT: alanine transaminase. Black: control, Blue: CM-272 treatment. (F) Liver H3K9me2 and 5mC levels after in vivo CM-272 treatment in CEMO-1 ALL mouse model. H3 total was used as a loading control. (G) Mean ± SEM body weight of mice engrafted with CEMO-1 after 28 days of treatment with CM-272. Black: control (n=6), Blue: CM-272 treatment (n=6). (H) Mean ± SEM body weight of mice engrafted with MV4-11 after 21 days of treatment with CM-272. Black: control (n=6), Blue: CM-272 treatment (n=6). (I) Mean ± SEM body weight of mice engrafted with OCI-Ly10 after 8 weeks of treatment with CM-272. Black: control (n=6), Blue: CM-272 treatment (n=6). Error bars indicate s.d. from three replicates.

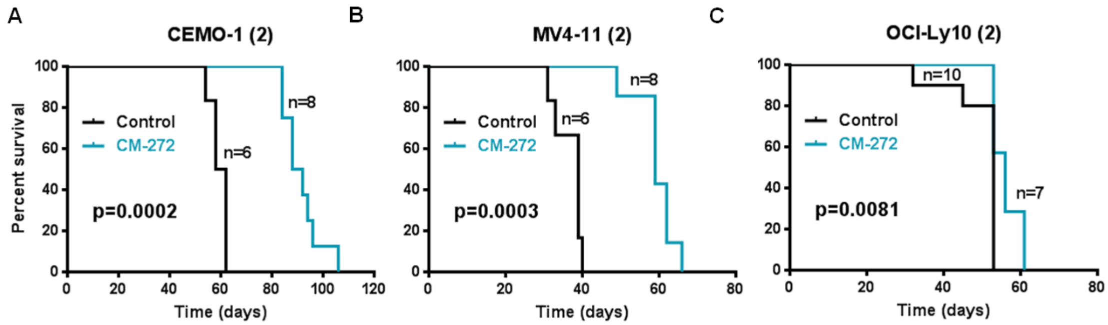

Supplementary Figure 11: CM-272 shows anti-leukemic effects in vivo. Kaplan-Meier survival curves for evaluating the survival time of mice engrafted with ALL derived CEMO-1 cells (A), AML derived MV4-11 (B) and DLBCL derived OCI-Ly10 (C) treated with CM-272. Control: Saline Solution (diluent of CM-272). *P* assessed by log-rank.

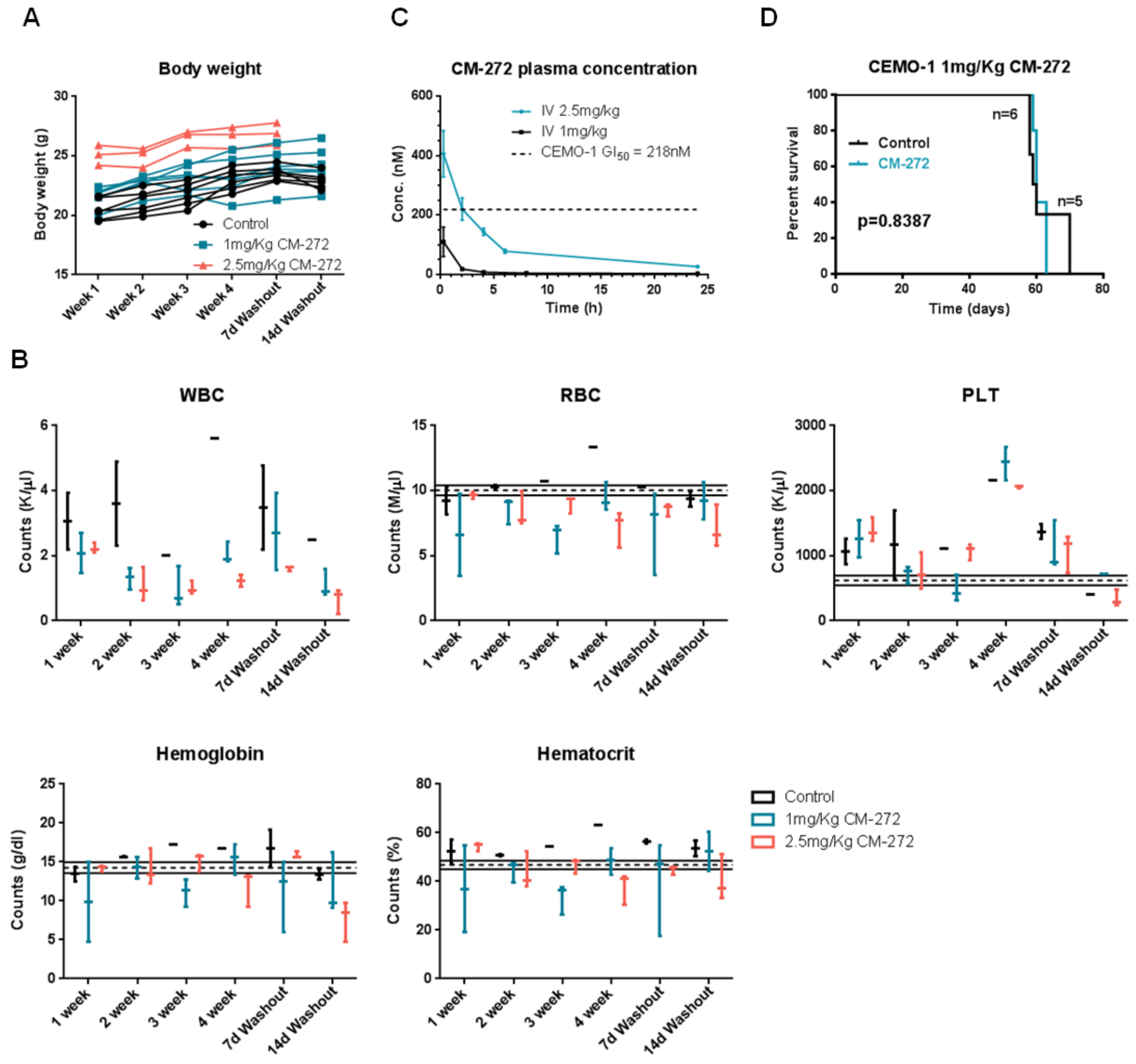

Supplementary Figure 12. CM-272 is dose-dependent *in vivo*. (A) Body weight in *in vivo* ALL model. Mean ± SEM body weight in CEMO-1 mice treated with Control (Saline Solution), 1 or 2.5 mg/kg daily for 4 weeks followed by a day's washout period. (B) Hematological parameters in *in vivo* CEMO-1 ALL model treated with 1 or 2.5 mg/kg of CM-272. WBC: white blood cells; RBC: red blood cells; PLT: platelet count. Black: Control group with saline solution, Blue: 1 mg/kg CM-272 treatment, Salmon: 2.5 mg/kg CM-272 treatment. (C) CM-272 plasma concentration in the CEMO-1 ALL treated with 1 mg/kg or 2.5 mg/kg of CM-272 and measured for four weeks plus 14 days of washout period. (D) Kaplan-Meier survival curves for evaluating the survival time of mice engrafted with ALL derived CEMO-1 cells treated with 1 mg/kg of CM-272. Control: Saline Solution (diluent of CM-272). Error bars indicate s.d. from three replicates.

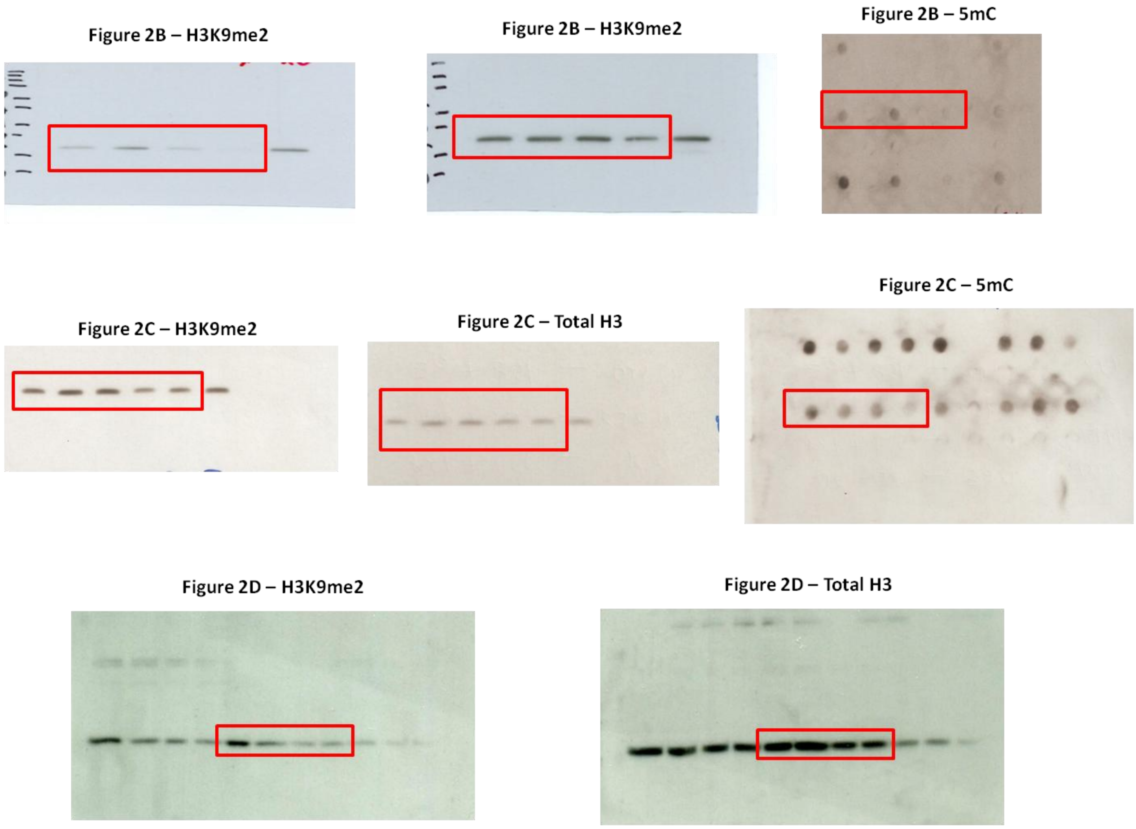

Supplementary Figure 13. Raw gels of Figure 2.

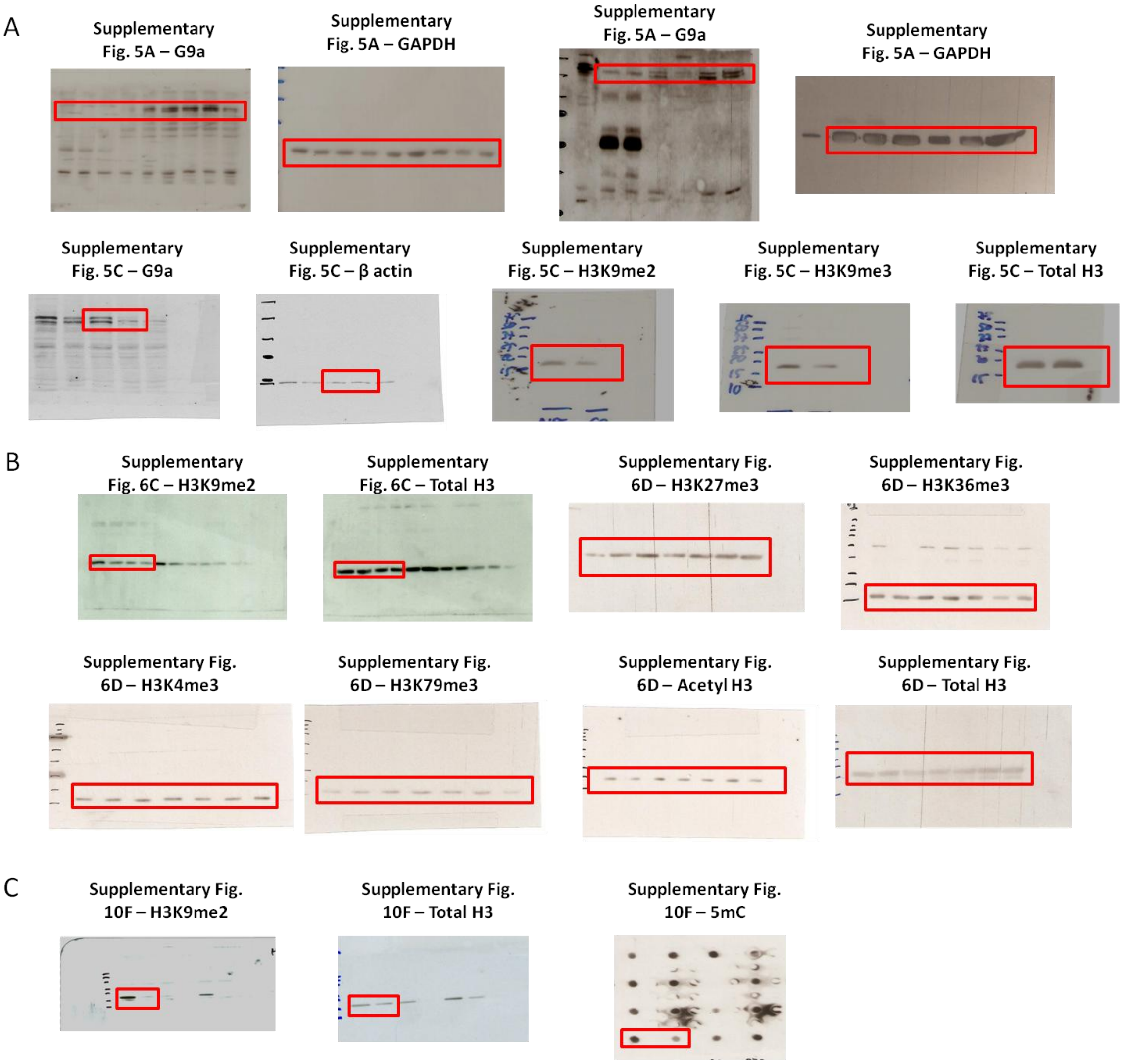

Supplementary Figure 14. Raw gels of Supplementary Figure 5 (A), 6 (B) and 10 (C).

Supplementary Tables

Supplementary Table 1: ADME and Cardiovascular safety profiles of CM-272 and CM-579

**ADME**		
	**CM-272**	**CM-579**
P450s Inhibition:<br>1A2, 2C19, 2C9, 2D6, 3A4	All with inhibition < 10% at 10µM	All with inhibition < 10% at 10µM
Plasma Protein Binding.<br>% Unbound:	24.4% (Human)<br>25.0% (Mouse)	14.7% (Human)<br>4.6% (Mouse)
Solubility (at pH=7.4):	117.6 µg/mL	>144.1 µg/mL
PAMPA (Pe, 10^{-6} in nm/s):	0.16	0.01
Liver Microsomal Stability.<br>Estimation $T_{1/2}$ (min)	>145 (Human)<br>35.2 (Mouse)	88.8 (Human)<br>10.7 (Mouse)
**Cardiovascular Safety**		
	**CM-272**	**CM-579**
hERG binding	IC_{50}: >100 µM	IC_{50}: >100 µM

Supplementary Table 2: LC_{50} values on cytotoxicity assays

Cell Type	CM-272 LC_{50} (μM)	CM-579 LC_{50} (μM)
THLE-2	1.78	1.30
PBMCs	1.90	7.39

Supplementary Table 3: IC_{50} (nM) against G9a and DNMT1 and GI_{50} (nM) against different cell lines of selected compounds shown in Figure 1b.

	G9a IC_{50}	DNMT1 IC_{50}	CEMO-1	LAL-CUN-2	OCI-AML-2	MV4-11	OCI-Ly3	OCI-Ly10
**BIX-01294**	1200[b]	>10000[d]	2910	5940	6320	11900	>4000	>4000
**UNC-0638**	55[c]	2010[e]	4730	5180	7330	12000	>4000	>4000
**CM-272**	8[f]/2[g]	382[f]/84[g]	218	664	256	269	409	455
**CM-579**	16[f]/47[g]	32[f]/70[g]	75	365	490	385	202	<31
**Azacitidine**	>10000	300[a]	3960	21200	>10000	5560	1930	2600
**Decitabine**	>10000	30[a]	506	>22400	>10000	>50000	>4000	>4000

[a] Data taken from Foulks et al [2].

[b] Internally determined data, in agreement with reported IC_{50} value of 1900 nM [3].

[c] Internally determined data, in agreement with reported IC_{50} value of <15 nM [4].

[d] Internally determined data.

[e] Internally determined data, in agreement with reported IC_{50} value of 1287 nM as reported at http:/thesgc.org/chemical-probes/UNC0638

[f] Internally determined data, according to protocol described above

[g] Externally determined data, using a radioligand binding assay (^{3}H-SAM, tritiated SAM), performed by www.reactionbiology.com/

Supplementary Table 4:

A) Percentage of inhibition of compounds CM-272 and CM-579 at 10 μM against a pannel of 37 epigenetic targets.

TARGET	CM-272	CM-579	Family
EZH1	6	17	HMT
EZH2	1	0	HMT
GLP	100	100	HMT
MLL-WARD	4	2	HMT
PRMT1	73	43	HMT
PRMT3	7	5	HMT
PRMT4	5	6	HMT
PRMT5	6	0	HMT
PRMT6	82	40	HMT
PRMT8	11	12	HMT
SET7-9	3	0	HMT
SETD2	8	0	HMT
SUV39H1	1	2	HMT
SUV39H2	4	0	HMT
ATAD2A	0	0	BRD
ATAD2B	0	0	BRD
BAZ2B	0	0	BRD
BRD1	0	N.D.	BRD
BRD2(BD1+BD2)	0	0	BRD
BRD4(BD1+BD2)	56	0	BRD
BRDT(BD1)	8	0	BRD
CREBBP	5	0	BRD
TAF1	0	4	BRD
TRIM24	0	0	BRD
JMJD1A	8	6	HDMT
JMJD2A	1	30	HDMT
JMJD2B	2	40	HDMT
JMJD2C	12	13	HDMT
JMJD2D	5	25	HDMT
JMJD2E	1	8	HDMT
JMJD3	9	3	HDMT
Jarid1A	2	27	HDMT
Jarid1B	5	27	HDMT
Jarid1C	1	13	HDMT
LSD1	0	24	HDMT
HDAC1	0	0	HDAC
HDAC6	0	0	HDAC

HMT = Histone Methyltransferase; BRD = Bromodomains; HDMT = Histone Demethylase; HDAC = Histone Deacetylase
N.D. Not determined

B) IC_{50} values against different targets from the panel for epigenetics profiling with a percentage of inhibition higher than 50% at 10 μM. RBA = radioligand binding assay (^{3}H-SAM, tritiated SAM), performed by www.reactionbiology.com/

TARGET	CM-272	CM-579
DNMT3A (IC_{50}, nM)	85	92
DNMT3B (IC_{50}, nM)	1200	1000
GLP (IC_{50}, nM)	2 (7nM using RBA)	> 10000 (67nM using RBA)
PRMT1 (IC_{50}, nM)	4000	> 10000
PRMT6 (IC_{50}, nM)	3000	> 10000
BRD4-BD1-BD2 (IC_{50}, nM)	1500	> 10000

Supplementary Table 5: IC_{50} values for the G9a competition assay with SAM.

SAM (μM)	CM-272 IC_{50} (nM)	CM-579 IC_{50} (nM)
2	15.57	3.54
20	7.05	1.32
40	2.26	1.44
80	4.59	N.D.
160	< 0.80	1.68
320	3.54	1.24
640	5.13	1.12

N.D. Not determined

Supplementary Table 6: IC_{50} values for the G9a competition assay with PepMe1.

PepMe1 (nM)	CM-272 IC_{50} (nM)	CM-579 IC_{50} (nM)
40	6.579	1.20
160	10.42	5.70
320	13.27	7.67
640	28.56	14.49
1280	29.65	15.39
2560	41.74	26.27

Supplementary Table 7: IC_{50} values for the DNMT1 competition assay with SAM.

SAM (μM)	CM-272<br>IC_{50} (nM)	CM-579<br>IC_{50} (nM)
1	175	24.05
10	389	19.57
25	338	21.68
50	301	26.02
100	358	25.18

Supplementary Table 8: IC_{50} values for the DNMT1 competition assay with pdI-pdC.

pdI-pdC (μg/mL)	CM-272 IC_{50} (nM)	CM-579 IC_{50} (nM)
1	143	21.43
3	188	56.47
10	713	171
30	1526	473
100	5968	2954

Supplementary Table 9: CM-272 plasmatic concentrations after intravenous (i.v.) administration (2.5 mg/kg) (n= 4). BLOQ: below limit of quantification.

**Time (h)**	**[CM-272] (nM)**	
	**Mean**	**SD**
0.25	406	78.3
2	220	37.6
4	143	12.2
8	123	7.6
24	BLOQ	-

Supplementary Table 10: CM-579 plasmatic concentrations after intravenous (i.v.) administration (1 mg/kg) (n= 4).

Time (h)	[CM-579] (nM)	
	**Mean**	**SD**
0.25	190.6	109.3
1	38.5	25.1
2	19.2	15.4
4	16.6	9.7
8	10.0	6.1

Supplementary Table 11: CM-272 and CM-579 pharmacokinetic parameters estimated by fitting previously reported experimental data (Supplementary Tables 11 and 12) to a non-compartmental model (NCA) using Winnonlin software for pharmacokinetic analysis. The parameters are: Area Under the Curve computed to the last observation (AUClast); Maximum concentration (Cmax); half life of the product (T½); clearance (Cl); volume of distribution (Vss).

**Parameter**	**CM-272 Mean value**	**CM-579 Mean value**
**AUClast (nM*h)**	**1494**	**268.4**
**Cmax (nM)**	**406**	**190.6**
**$T^{1/2}$ (h)**	**24.2**	**6.26**
**Cl (L/h/kg)**	**0.91**	**5.7**
**Vss (L)**	**0.73**	**0.81**
**Norm. Vss (L/kg)**	**29.3**	**32.4**

Supplementary Table 12. Selectivity profiling of CM-272 (10 μM) against a panel of 97 Kinases. Results for primary screen binding interactions are reported as 'Percent Control', with lower values corresponding to stronger hits.

DiscoveRx Gene Symbol	Entrez Gene Symbol	Percent Control
ABL1(E255K)-phosphorylated	ABL1	5.5
ABL1(T315I)-phosphorylated	ABL1	25
ABL1-nonphosphorylated	ABL1	9.4
ABL1-phosphorylated	ABL1	4.8
ACVR1B	ACVR1B	81
ADCK3	CABC1	100
AKT1	AKT1	100
AKT2	AKT2	100
ALK	ALK	0.5
AURKA	AURKA	71
AURKB	AURKB	61
AXL	AXL	100
BMPR2	BMPR2	32
BRAF	BRAF	14
BRAF(V600E)	BRAF	27
BTK	BTK	0.5
CDK11	CDK19	88
CDK2	CDK2	100
CDK3	CDK3	100
CDK7	CDK7	53
CDK9	CDK9	96
CHEK1	CHEK1	99
CSF1R	CSF1R	100
CSNK1D	CSNK1D	95
CSNK1G2	CSNK1G2	95
DCAMKL1	DCLK1	22
DYRK1B	DYRK1B	100
EGFR	EGFR	100
EGFR(L858R)	EGFR	100
EPHA2	EPHA2	100
ERBB2	ERBB2	0
ERBB4	ERBB4	97
ERK1	MAPK3	100
FAK	PTK2	96
FGFR2	FGFR2	100
FGFR3	FGFR3	100
FLT3	FLT3	100
GSK3B	GSK3B	61
IGF1R	IGF1R	97
IKK-alpha	CHUK	45
IKK-beta	IKBKB	13
INSR	INSR	5.1
JAK2(JH1domain-catalytic)	JAK2	10
JAK3(JH1domain-catalytic)	JAK3	0
JNK1	MAPK8	25

JNK2	MAPK9	34
JNK3	MAPK10	8.9
KIT	KIT	100
KIT(D816V)	KIT	100
KIT(V559D,T670I)	KIT	100
LKB1	STK11	100
MAP3K4	MAP3K4	93
MAPKAPK2	MAPKAPK2	87
MARK3	MARK3	100
MEK1	MAP2K1	45
MEK2	MAP2K2	22
MET	MET	91
MKNK1	MKNK1	1.4
MKNK2	MKNK2	18
MLK1	MAP3K9	100
p38-alpha	MAPK14	96
p38-beta	MAPK11	92
PAK1	PAK1	69
PAK2	PAK2	67
PAK4	PAK4	100
PCTK1	CDK16	23
PDGFRA	PDGFRA	14
PDGFRB	PDGFRB	100
PDPK1	PDPK1	100
PIK3C2B	PIK3C2B	61
PIK3CA	PIK3CA	40
PIK3CG	PIK3CG	88
PIM1	PIM1	93
PIM2	PIM2	93
PIM3	PIM3	81
PKAC-alpha	PRKACA	100
PLK1	PLK1	31
PLK3	PLK3	73
PLK4	PLK4	80
PRKCE	PRKCE	0
RAF1	RAF1	73
RET	RET	100
RIOK2	RIOK2	27
ROCK2	ROCK2	0.3
RSK2(Kin.Dom.1-N-terminal)	RPS6KA3	46
SNARK	NUAK2	12
SRC	SRC	100
SRPK3	SRPK3	100
TGFBR1	TGFBR1	78
TIE2	TEK	90
TRKA	NTRK1	0
TSSK1B	TSSK1B	100
TYK2(JH1domain-catalytic)	TYK2	22
ULK2	ULK2	9.8
VEGFR2	KDR	23

YANK3	STK32C	100
ZAP70	ZAP70	27

Supplementary Table 13. Selectivity Score of CM-272 against 90 non-mutant kinases from the KINOME*scan profiling.*

Selectivity Score Type	Number of Hits	Selectivity Score
S(35)	29	0.322
S(10)	12	0.133
S(1)	7	0.078

Number of hits corresponds to the number of non-mutant kinases with Percent Control (Supplementary Table 12) lower than the indicated threshold (e.g. S(35) corresponds to a Percent Control < 35%). Selectivity Score (S-score) is calculated by dividing the number of kinases that compounds bind to by the total number of distinct kinases tested, excluding mutant variants (90 in this assay).

Supplementary Table 14. K_d (nM) values of CM-272 against 7 selected kinases from the total panel, having Percent Control < 1%.

Entrez Gene Symbol	Kd (nM)
ALK	820
BTK	1100
ERBB2	1600
JAK3 (JH1domain-catalytic)	1800
PRKCE	1200
ROCK2	1700
TRKA	1100

K_d determination was also performed by DiscoverRx (http://www.discoverx.com/home) using the KINOME*scan*™ screening platform (*1*). KINOMEscan are heterogeneous competitive active-site binding assays (not based on catalytic activity) and do not employ ATP (substrate concentration is zero). Thus considering Cheng-Prusoff equation, in this case [1 + (0/Km)] = 1, $K_d = IC_{50}$. According to PK data, where Cmax for CM-272 is 406 nM (Supplementary Table 11), compound concentration at administered dose is below the required IC_{50} values to inhibit these kinases.

Supplementary Methods

G9a and DNMT1 docking

CM-272 was superposed to the conformation of UNC0638 in the co-crystal structure of the G9-UNC0638-SAH complex (Protein Data Bank, PDB, entry 3RJW) with the MOE program (Chemical Computing Group, http://www.chemcomp.com/). Then, the overlaid conformation of CM-272 was translated into the G9a-UNC0638-SAH crystal in order to analyze the key interactions between the ligand and the methyltransferase.

The GoldSuite 5.2 program (Cambridge Crystallographic Data Centre, https://www.ccdc.cam.ac.uk/pages/Home.aspx) was used to carry out docking of CM-272 to DNMT1. The crystal structure of Mouse DNMT1 bound to hemimethylated CpG DNA (PDB entry 4DA4) was chosen. The docking region used was a 20-Å sphere around the carboxylate oxygen of Glu1269. The PLP scoring function was used to rank docking poses, and protein hydrogen bond constraints for binding to carboxylate of Glu-1269 were imposed on the ligand. The top twenty best-docked structures out of 100 independent genetic algorithm runs were retrieved and visually inspected. The high-scoring pose was finally chosen as it has a plausible binding mode with key interactions with DNMT1 and a high degree of convergence (r.m.s.d < 2 Å) was observed among the top three ranked poses.

General Chemistry Information.

The NMR spectroscopic data were recorded on a Bruker AV400 or VARIAN 400MR spectrometer. Chemical shifts (δ) are reported in parts per million (ppm). The abbreviations used to explain multiplicities are s = singlet, d = doublet, t = triplet, m = multiplet. Coupling constants (*J*) are in hertz. Melting points were determined on a Mettler FP82 hot stage controlled by a Mettler FP80 central processor.

High Resolution Mass Spectrometry of final compounds

High resolution mass spectrometry (HRMS) *m/z* determination was performed by an Agilent Technologies 1200 liquid chromatographic system equipped with a 6220 Accurate-Mass TOF LC/MS, operated in positive electrospray ionization mode (ESI+) controlled by MassHunter Workstation 06.00 software (Agilent Technologies, Barcelona, Spain). Separation was carried out on a Zorbax SB-C18 (15 cm × 0.46 cm; 5 µm) from Agilent Technologies with a SB-C18 precolumn from Teknokroma (Barcelona, Spain). Solvent A: water with 0.1% formic acid; Solvent B: acetonitrile. The gradient elution was 5 % B, 0-0.5 min; 5-100 % B 0.5-8.0 min; 100-5 % B 8.0-10.0 min. The injection volume was 10 µL and the flow rate was 0.5 mL min−1. Chromatography was performed at 40 ºC. ESI conditions were as follows: gas temperature, 350 ºC; drying gas, 10 L min−1 ; nebulizer, 45 psig; capillary voltage, 3500 V; fragmentor, 175 V; and skimmer, 65 V. The instrument was set out to acquire over the m/z range 100 –1000 with an acquisition rate of 1.03 spectra s-1.

Synthesis of compound CM-272

Protocol for preparative HPLC purification method

The HPLC measurement was performed using Gilson 281 from 233 pump (binary), an auto-sampler and a UV detector. The fractions were detected by LC-MS. The MS detector was configured with an electrospray ionization source. The source temperature was maintained at 300-350 ºC. Reverse phase HPLC was carried out on Luna C18 (100×30 mm; 4µm). Solvent A: water with 0.075% trifluoroacetic acid; Solvent B: acetonitrile with 0.075% trifluoroacetic acid. Gradient: At room temperature, 20% of B to 40% of B within 6 minutes at 25 mL/min; then 40% B at 25 mL/min over 2 minutes.

Protocol for HPLC-analysis

HPLC-analysis was performed using a Shimadzu LC-20AB or LC-20AD with a Luna-C18 (2), 5µm, 2.0*50mm column at 40 ºC and UV detection. Solvent A: water with 0.056% trifluoroacetic acid; Solvent

B: acetonitrile with 0.056% trifluoroacetic acid. Gradient: After 0.1 minutes at the initial condition of 90% A and 10% B, solvent B was increased to 80% over 4 minutes, maintained at 80% for 0.9 minutes. Then a linear gradient to initial conditions was applied for 0.02 minutes and maintained for 0.58 minutes to re-equilibrate the column, giving a cycle time of 5.50 minutes. Flow rate was 0.8 mL/min from 0.01 to 4.90 minutes, increased to 1.2 mL/min in 0.03 minutes and maintained until the end of the run.

Synthesis of compound CM-272

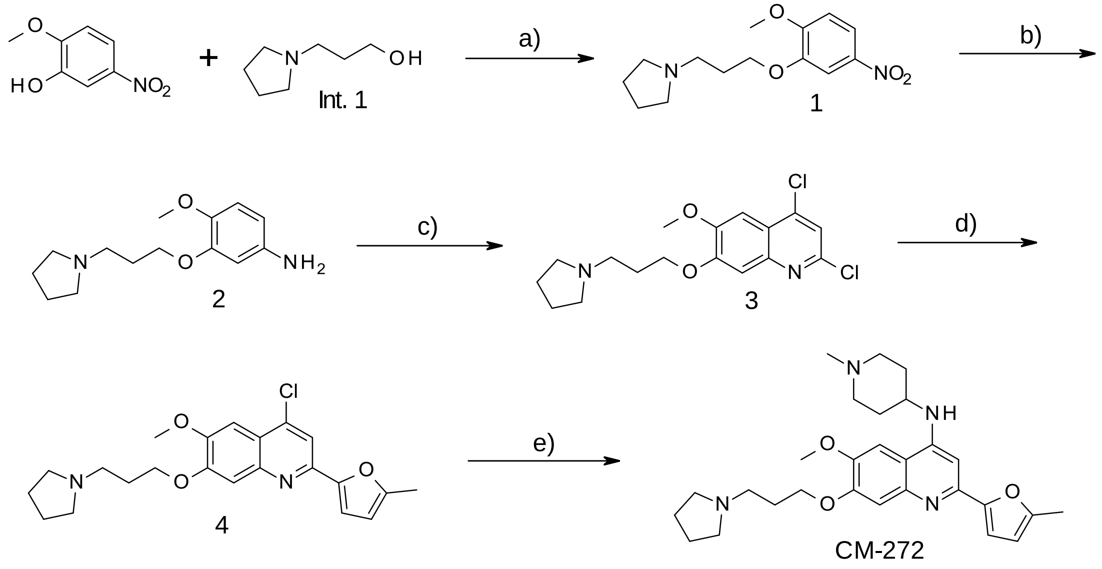

Conditions: a) **Int. 1**, PPh_3, DEAD, THF, 0 ºC, then rt, 5 h; b) Pd/C, H_2 (1 atm), MeOH, rt, 3 h; c) malonic acid, $POCl_3$, rt, 4 h, then 90 ºC, overnight; d) 4,4,5,5-tetramethyl-2-(5-methyl-2-furyl)-1,3,2-dioxaborolane, $Pd(PPh_3)_4$, Na_2CO_3, 1,4-dioxane/H_2O (5:1), 110 ºC, MW, 2 h; e) 1-methylpiperidin-4-amine, $Pd_2(dba)_3$, BINAP, Cs_2CO_3, 1,4-dioxane, 110 ºC, MW, 2 h.

*Preparation of 3-pyrrolidin-1-ylpropan-1-ol (**Int. 1**)*: To a solution of commercially available pyrrolidine (20 g, 0.28 mol) in THF (300 mL) was added 3-bromopropan-1-ol (46 g, 0.34 mol) and K_2CO_3 (74 g, 0.54 mol) and the mixture was stirred at 70 ºC overnight. Then, the reaction mixture was concentrated under

vacuum. The residue was extracted with CH_2Cl_2 and washed with water. The organic layer was concentrated to give the desired product Int. 1 (28 g, 77%). MS *m/z* 130 [(M + H)]+ calcd. for $C_7H_{15}NO$.

*Preparation of 1-[3-(2-methoxy-5-nitro-phenoxy)propyl]pyrrolidine (**1**):* To a solution of commercially available 2-methoxy-5-nitro-phenol (10 g, 0.06 mol) in THF (100 mL) was added PPh_3 (37.5 g, 0.14 mol), Int. 1 (7.3 g, 0.06 mol, prepared as described above) and DEAD (40 g, 0.23 mol) at 0 ºC and the solution was stirred at room temperature for 5 hours. Then, the reaction mixture was concentrated and extracted with EtOAc. The combined organic layers were washed with brine, dried over anhydrous Na_2SO_4 and concentrated to give the crude product which was purified by column chromatography to obtain compound 1 (9 g, 54%) as yellow solid. MS *m/z* 281 [(M + H)]+ calcd. for $C_{14}H_{20}N_2O_4$.

*Preparation of 4-methoxy-3-(3-pyrrolidin-1-ylpropoxy)aniline (**2**):* To a solution of compound 1 (9 g, 0.032 mol) in MeOH (200 mL) was added Pd/C (5 g) and the solution was stirred at room temperature for 3 hours under H_2 atmosphere (1 atm). Then, the solution was filtered and the filtrate was concentrated to give compound 2 (7 g, 87%) as yellow oil. MS *m/z* 251 [(M + H)]+ calcd. for $C_{14}H_{22}N_2O_2$.

*Preparation of 2,4-dichloro-6-methoxy-7-(3-pyrrolidin-1-ylpropoxy)quinoline (**3**)*: To a solution of compound 2 (7 g, 0.028 mol) in $POCl_3$ (40 mL) was added malonic acid (5.7 g, 0.055 mol) at room temperature. After stirring at room temperature for 4 hours, the solution was heated to 90 ºC overnight. Then, the solution was concentrated and poured into ice-water. The mixture was extracted with EtOAc and the combined organic layers were washed with brine, dried over anhydrous Na_2SO_4 and concentrated to give the compound 3 (3.5 g, 35%) as pale yellow solid. MS *m/z* 355 [(M + H)]+ calcd. for $C_{17}H_{20}Cl_2N_2O_2$.

*Preparation of 4-chloro-6-methoxy-2-(5-methyl-2-furyl)-7-(3-pyrrolidin-1-ylpropoxy)quinoline (**4**):* To a solution of compound 3 (1 g, 2.8 mmol) in 1,4-dioxane/H_2O (5:1, 18 mL) was added Na_2CO_3 (1 g, 9.4 mmol), $Pd(PPh_3)_4$ (0.32 g, 0.28 mmol) and 4,4,5,5-tetramethyl-2-(5-methyl-2-furyl)-1,3,2-dioxaborolane (0.64 g, 3.08 mmol). The solution was heated at 110 ^oC for 2 hours under Microwave. Then, the reaction was quenched with water and extracted with EtOAc. The organic layer was washed with brine, dried over anhydrous Na_2SO_4 and concentrated to give the crude product 4 which was purified by preparative TLC to obtain compound 4 (500 mg, 45%) as a yellow solid. MS *m/z* 401.2 [(M + H)]+ calcd. for $C_{22}H_{25}ClN_2O_3$.

*Preparation of 6-methoxy-2-(5-methyl-2-furyl)-N-(1-methyl-4-piperidyl)-7-(3-pyrrolidin-1-ylpropoxy)quinolin-4-amine (**CM-272**):* To a solution of compound 4 (500 mg, 1.25 mmol) in 1,4-dioxane (10 mL) was added Cs_2CO_3 (1.2 g, 3.75 mmol), BINAP (0.2 g, 0.32 mmol), $Pd_2(dba)_3$ (0.18 g, 0.20 mmol) and 1-methylpiperidin-4-amine (285 mg, 2.5 mmol) and the solution was heated at 110 ºC for 2 hours under Microwave. Then, the mixture was quenched with water and extracted with EtOAc. The organic layer was washed with brine, dried over anhydrous Na_2SO_4 and concentrated to give the crude product which was purified by preparative HPLC (General procedure described above) to obtain pure compound CM-272 (87.6 mg, 15%). Purity is 99.65% according to HPLC analytical method (described above); where Rt is 1.54. m.p. 180-181 ºC. ^1H NMR (MeOD, 400 MHz): δ 7.81 (s, 1H), 7.58 (d, *J* = 2.8 Hz 1H), 7.50 (s, 1H), 7.09 (s, 1H), 6.44 (s, 1H), 4.35 (m, 4H), 4.04 (s, 3H), 3.83 (m, 2H), 3.72 (m, 2H), 3.51 (m, 2H), 3.27 (m, 1H), 3.17 (m, 2H), 2.95 (s, 3H), 2.51 (s, 3H), 2.40 (m, 4H), 2.21 (m, 6H). ^{13}C NMR (DMSO-d_6, 100 MHz): δ 157.0, 153.0, 152.9, 148.7, 144.0, 139.6, 134.6, 116.5, 110.3, 110.0, 102.7, 101.2, 92.1, 66.1, 56.6, 53.2 (2C), 52.5 (2C), 51.4, 47.7, 42.7, 28.5 (2C), 25.0, 22.6 (2C), 13.6. HRMS *m/z*: $[(M+H)]^+$ calcd. for $C_{28}H_{38}N_4O_3$: 479.3022; found, 479.3060.

Synthesis of compound CM-579

Protocol for preparative HPLC purification method

The HPLC measurement was performed using Gilson 281 from 233 pump (binary), an auto-sampler and a UV detector. The fractions were detected by LC-MS. The MS detector was configured with an electrospray ionization source. The source temperature was maintained at 300-350 ºC. Reverse phase HPLC was carried out on Luna C18 (100×30 mm; 4µm). Solvent A: water with 0.075% trifluoroacetic acid; Solvent B: acetonitrile with 0.075% trifluoroacetic acid. Gradient: At room temperature, 25% of B to 45% of B within 6 minutes at 20 mL/min; then 40% B at 25 mL/min over 3 minutes.

Protocol for LCMS-analysis

LCMS-analysis was performed using an Agilent 1200, 6120MSD Quadrupole detector with a Luna-C18 (2.0 x 50 mm, 5µm) column at 50 ºC and UV detection (220 nm). Solvent A: water with 0.037% trifluoroacetic acid; Solvent B: acetonitrile with 0.019% trifluoroacetic acid. Gradient: After 0.4 minutes at the initial condition of 99% A and 1% B, solvent B was increased to 90% over 3 minutes. Then solvent B was increased to 100% over 0.45 minutes. Finally a linear gradient to initial conditions was applied for 0.01 minutes to re-equilibrate the column. Flow rate was 0.8 mL/min.

Synthesis of CM-579

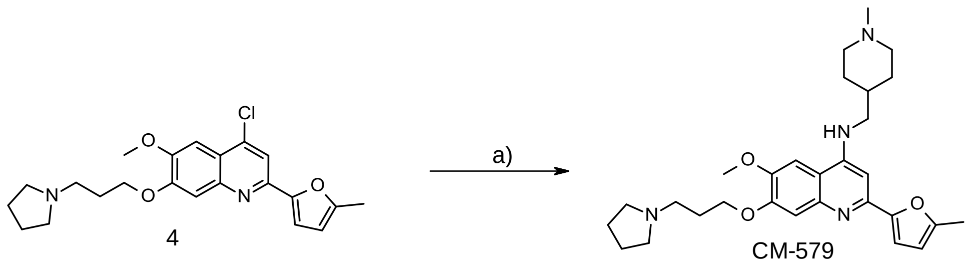

Conditions: a) (1-methylpiperidin-4-yl)methanamine , $Pd_2(dba)_3$, BINAP, Cs_2CO_3, 1,4-dioxane, 120 ºC, MW, 3 h.

*Preparation of 6-methoxy-2-(5-methyl-2-furyl)-N-[(1-methyl-4-piperidyl)methyl]-7-(3-pyrrolidin-1-ylpropoxy)quinolin-4-amine (**CM-579**):* To a solution of compound 4 (200 mg, 0.5 mmol) in 1,4-dioxane (10 mL) was added Cs_2CO_3 (325 mg, 1 mmol), BINAP (67 mg, 0.1 mmol), $Pd_2(dba)_3$ (30 mg, 0.03 mmol) and (1-methylpiperidin-4-yl)methanamine (230 mg, 1.8 mmol) and the solution was heated at 120 ºC for 3 hours under Microwave. Then, the solution was concentrated and extracted with EtOAc. The combined organic layer was washed with brine, dried over anhydrous Na_2SO_4 and concentrated to give the crude product which was purified by preparative HPLC (General procedure described above) to give CM-579 (60 mg, 24%) as yellow solid. Purity is 98.37% according to LCMS analytical method (described above); where Rt is 2.06. m.p. 34-35 ºC. ^{1}H NMR (MeOD, 400 MHz): δ 7.73 (s, 1H), 7.55 (d, J = 2.4 Hz, 1H), 7.48 (s, 1H), 7.00 (s, 1H), 6.42 (d, J = 2.4 Hz, 1H), 4.35 (m, 2H), 4.03 (s, 3H), 3.82 (m, 2H), 3.62-3.56 (m, 4H), 3.51-3.47 (m, 2H), 3.17 (m, 2H), 3.03 (m, 2H), 2.86 (s, 3H), 2.50 (s, 3H), 2.39-2.36 (m, 2H), 2.21-2.06 (m, 7H), 1.65 (m, 2H). ^{13}C NMR (DMSO-d_6, 100 MHz): δ 156.8, 153.9, 153.0, 148.7, 143.9, 139.4, 134.4, 116.3, 110.2, 109.9, 102.4, 101.2, 91.9, 66.1, 56.3, 53.2 (2C), 53.0 (2C), 51.3, 47.1, 42.6, 32.1, 26.9 (2C), 25.0, 22.6 (2C), 13.6. HRMS *m/z*: $[(M+H)]^+$ calcd. for $C_{29}H_{40}N_4O_3$, 493.3179; found, 493.3178.

Synthesis of compound CM-1021

Protocol for preparative HPLC purification method

The HPLC measurement was performed using Gilson 281 from 233 pump (binary), an auto-sampler and a UV detector. The fractions were detected by LC-MS. The MS detector was configured with an electrospray ionization source. The source temperature was maintained at 300-350 ºC. Reverse phase HPLC was carried

out on Luna C18 (100×30 mm; 4µm). Solvent A: water with 0.075% trifluoroacetic acid; Solvent B: acetonitrile with 0.075% trifluoroacetic acid. Gradient: At room temperature, 25% of B to 45% of B within 6 minutes at 20 mL/min; then 40% B at 25 mL/min over 3 minutes.

Protocol for LCMS-analysis

LCMS-analysis was performed using an Agilent 1200, 6120MSD Quadrupole detector with a Luna-C18 (2.0 x 50 mm, 5µm) column at 50 ºC and UV detection (220 nm). Solvent A: water with 0.037% trifluoroacetic acid; Solvent B: acetonitrile with 0.019% trifluoroacetic acid. Gradient: After 0.4 minutes at the initial condition of 90% A and 10% B, solvent B was increased to 100% over 3 minutes and maintained at 100% for 0.45 minutes. Then a linear gradient to initial conditions was applied for 0.01 minutes to re-equilibrate the column. Flow rate was 0.8 mL/min.

Synthesis of CM-1021

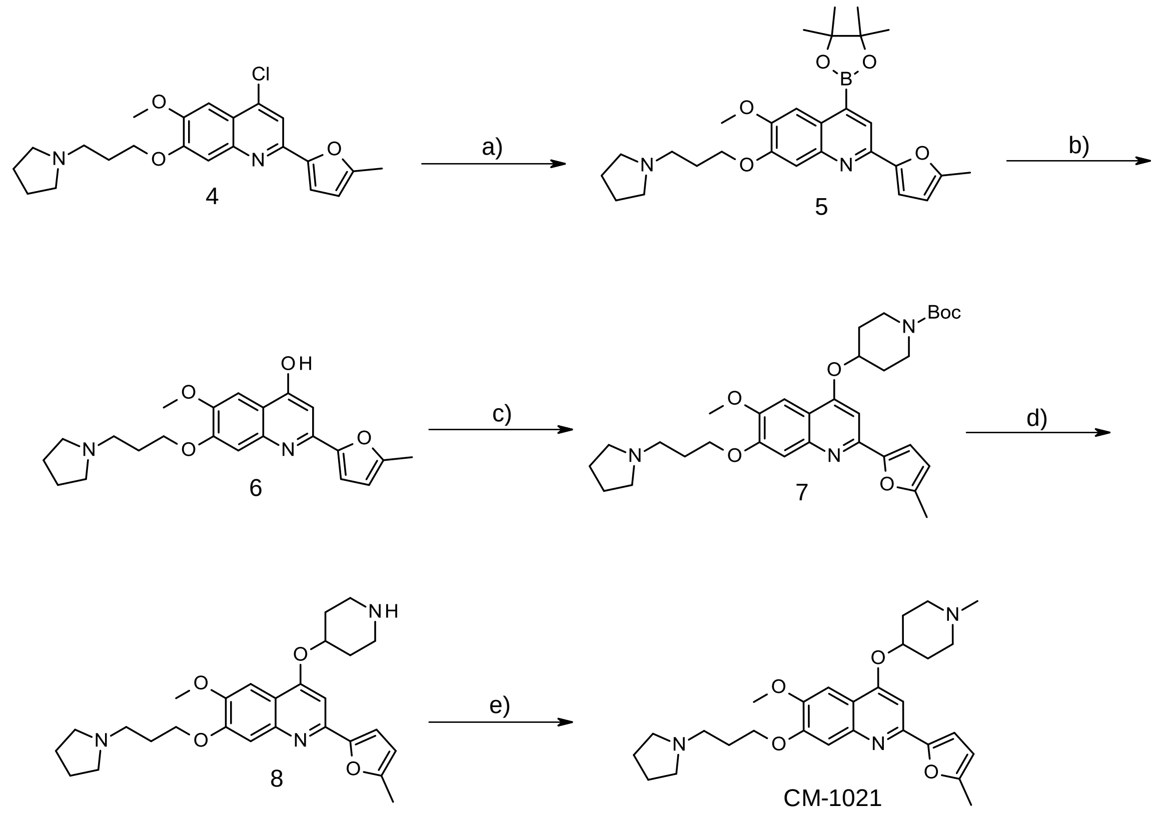

Conditions: a) 4,4,5,5-tetramethyl-2-(4,4,5,5-tetramethyl-1,3,2-dioxaborolan-2-yl)-1,3,2-dioxaborolane, KOAc, Pd(dppf)Cl_2, 1,4-dioxane, 100 ºC, 16 h; b) H_2O_2, CH_2Cl_2, 15 ºC, 16 h; c) *tert*-butyl 4-methylsulfonyloxypiperidine-1-carboxylate, Cs_2CO_3, DMF, 80 ºC, 16 h; d) HCl/EtOAc (2.0 M), 15 ºC, 5 h; e) $(HCHO)_n$, NaBH$(OAc)_3$, HCOOH, MeOH, 70 ºC, 16 h.

*Preparation of 6-methoxy-2-(5-methyl-2-furyl)-7-(3-pyrrolidin-1-ylpropoxy)-4-(4,4,5,5-tetramethyl-1,3,2-dioxaborolan-2-yl)quinoline (**5**):* A mixture of compound 4 (300 mg, 0.75 mmol), 4,4,5,5-tetramethyl-2-(4,4,5,5-tetramethyl-1,3,2-dioxaborolan-2-yl)-1,3,2-dioxaborolane (209 mg, 0.82 mmol), KOAc (183 mg, 1.87 mmol) and Pd(dppf)Cl_2 (109 mg, 0.15 mmol) in 1,4-dioxane (20 mL) was degassed and purged with N_2 for 3 times. Then, the mixture was stirred at 100 °C for 16 hours. Then, the reaction mixture was concentrated in vacuum to give a residue, which was purified by silica gel chromatography to obtain pure compound 5 (350 mg, 95%) as a black solid. MS *m/z* 493.4 [(M + H)]+ calcd. for $C_{28}H_{37}BN_2O_5$.

*Preparation of 6-methoxy-2-(5-methyl-2-furyl)-7-(3-pyrrolidin-1-ylpropoxy)quinolin-4-ol (**6**):* To a solution of compound 5 (350 mg, 0.71 mmol) in CH_2Cl_2 (15 mL) was added H_2O_2 (241 mg, 7.09 mmol) and the mixture was stirred at 15 °C for 16 hours. Then, the reaction mixture was poured into water and extracted with CH_2Cl_2. The organic layers was washed with saturated Na_2SO_3 aqueous solution, checked by potassium iodide-starch test paper, dried over Na_2SO_4 and concentrated in vacuum to give compound 6 (300 mg, crude) which was used into the next step without further purification as a black solid. MS *m/z* 383.3 [(M + H)]+ calcd. for $C_{22}H_{26}N_2O_4$.

*Preparation of tert-butyl 4-[[6-methoxy-2-(5-methyl-2-furyl)-7-(3-pyrrolidin-1-ylpropoxy)-4-quinolyl]oxy]piperidine-1-carboxylate (**7**):* To a solution of compound 6 (300 mg, 0.79 mmol) and tert-butyl 4-methylsulfonyloxypiperidine-1-carboxylate (328 mg, 1.18 mmol) in DMF (10 mL) was added Cs_2CO_3 (766 mg, 2.35 mmol) and the mixture was stirred at 80 °C for 16 hours. Then, the reaction mixture was concentrated in vacuum to give a residue, which was purified by preparative TLC to obtain compound 7 (120 mg, 27%) as a yellow solid. MS *m/z* 566.4 [(M + H)]+ calcd. for $C_{32}H_{43}N_3O_6$.

*Preparation of 6-methoxy-2-(5-methyl-2-furyl)-4-(4-piperidyloxy)-7-(3-pyrrolidin-1-ylpropoxy)quinoline (**8**):* Compound 7 (120 mg, 0.21 mmol) was dissolved in HCl/EtOAc (2.0 M, 20 mL) and the mixture was stirred at 15 °C for 5 hours. Then, the reaction mixture was concentrated in vacuum to give crude compound 8 (100 mg, 94%, HCl salt) as a yellow solid, which was used into the next step without further purification. MS *m/z* 466.6 [(M + H)]+ calcd. for $C_{27}H_{35}N_3O_4$.

*Preparation of 6-methoxy-2-(5-methyl-2-furyl)-4-[(1-methyl-4-piperidyl)oxy]-7-(3-pyrrolidin-1-ylpropoxy)quinoline (**CM-1021**):* A mixture of compound 8 (50 mg, 0.1 mmol), $(HCHO)_n$ (29 mg, 322.17 µmol), $NaBH(OAc)_3$ (68 mg, 0.32 mmol) and HCOOH (5 mg, 0.1 mmol) in MeOH (5 mL) was degassed and purged with N_2 for 3 times. Then the mixture was stirred at 70 °C for 16 hours. Then, the reaction mixture was concentrated in vacuum to give a residue, which was purified by preparative HPLC (General procedure described above) to obtain compound CM-1021 (26.3 mg, 55%) as a yellow oil. Purity is 99.01% according to LCMS analytical method (described above); where Rt is 1.62. ^{1}H NMR (MeOD, 400 MHz): δ 7.78 (s, 1H), 7.69-7.57 (m, 3H), 6.51 (d, *J* = 2.8 Hz, 1H), 4.41-4.38 (m, 2H), 4.11-4.05 (m, 3H), 3.87-3.73 (m, 3H), 3.52-3.46 (m, 5H), 3.18-3.13 (m, 2H), 3.00-2.97 (m, 3H), 2.61-2.57 (m, 1H), 2.56 (s, 3H), 2.42-2.39 (m, 4H), 2.27-2.18 (m, 3H), 2.10-2.07 (m, 2H). ^{13}C NMR (DMSO-d_6, 100 MHz): δ 153.6, 149.6, 120.9, 118.0, 115.0, 114.4, 114.3, 112.1, 110.3, 101.3, 100.7, 96.9, 96.8, 66.3, 56.2, 56.0, 53.3 (2C), 51.4 (2C), 48.8, 42.5, 27.8, 26.4, 25.0, 22.6 (2C), 13.7. HRMS *m/z*: $[(M+H)]^+$ calcd. for $C_{28}H_{37}N_3O_4$, 480.2862; found, 480.2853.

G9a enzyme activity assay

The biochemical assay to measure G9a enzyme activity relies on time-resolved fluorescence energy transfer (TR-FRET) between europium cryptate (donor) and XL665 (acceptor). TR-FRET is observed when biotinylated histone monomethyl-H3K9 peptide is incubated with cryptate-labeled anti-dimethyl-histone H3K9 antibody (CisBio Cat# 61KB2KAE) and streptavidin XL665 (CisBio Cat#610SAXLA), after enzymatic reaction of G9a.

The assay was carried out during 1 hour at room temperature, in a final volume of 20 µL, with 0.2 nM human G9a enzyme, 40 nM biotinylated histone monomethyl-H3K9 peptide, 20 µM S-adenosylmethionine (SAM) and different final concentrations of tested compounds in assay buffer (50 mM Tris-HCl, 10 mM NaCl, 4 mM DTT, 0.01% Tween-20 pH 9). The final percentage of DMSO was 0.5%. After incubation, adding 150 nM of cryptate-labeled anti-dimethylhistone H3K9 antibody (final concentration of 0.75 nM) and 16 µM of streptavidin XL665 beads stopped time enzyme activity. After one hour of incubation at

room temperature, fluorescence was measured at 620 nm and 665 nm. A ratio (665 nm / 620 nm) was then calculated in order to minimize medium interferences. Positive control was obtained in the presence of the vehicle of the compounds. Negative control was obtained in the absence of G9a enzyme activity. Calculated IC_{50} values were determined using GraphPrism using 4-parameters inhibition curve. Results are the average of three replicates.

DNMT1 enzyme activity assay

The biochemical assay to measure DNMT1 enzyme activity relies on time-resolved fluorescence energy transfer (TR-FRET) between lumi4-Tb (donor) and d2 (acceptor) using the EPIgeneous methyltransferase assay (CisBio Cat#62SAHPEB). TR-FRET is observed when antibody specific to S-adenosylhomocysteine labeled with Lumi4-Tb (CisBio Cat#62SAHPEB) is incubated with d2-labeled S-adenosylhomocysteine. TR-FRET signal is inversely proportional to the concentration of SAH, product of DNMT1 enzyme activity, in the sample.

The assay was carried out during 15 minutes at 37 ºC, in a final volume of 20 µL, with 20 nM human DNMT1 enzyme, 1 µg/mL poly-deoxy inosine poly-deoxy cytosine (pdI-pdC) DNA, 1 µM S-adenosylmethionine (SAM) and different final concentrations of tested compounds in assay buffer (50 mM Tris-HCl, 1 mM EDTA, 1 mM DTT, 0.1% Triton X-100, 5% glycerol pH 7.5). The final percentage of DMSO was 0.5%. After incubation time enzyme activity was stopped by adding 2 µL of buffer one of the EPIgeneous methyltransferase kit assay. After 10 minutes at room temperature, it was added 4 µL of antibody specific to S-adenosylhomocysteine labeled with Lumi4-Tb 50 x (dilution 1/50) and 4 µL of d2-labeled S-adenosylhomocysteine 31 x both diluted in buffer two of the EPIgeneous methyltransferase kit. Fluorescence was measured at 620 nm and 665 nm one hour later. A ratio (665 nm / 620 nm) was then calculated in order to minimize medium interferences. Positive control was obtained in the presence of the vehicle of the compounds. Negative control was obtained in the absence of DNMT1 enzyme activity. Calculated IC_{50} values were determined using GraphPrism using 4-parameters inhibition curve. Results are the average of three replicates.

Epigenetics Selectivity Pannel

Selectivity of CM-272 and CM-579 against 37 epigenetic targets Bromodomains (ATAD2A, ATAD2B, BAZ2B, BRD1, BRD2(BD1+BD2), BRD4(BD1+BD2), BRDT(BD1), CREBBP, TRIM24, TAF1), Histone Methyltransferases (EZH1, EZH2, GLP, PRMT1, PRMT3, PRMT4, PRMT5, PRMT6, PRMT8, SETD2, SET7/9, SUV39H1, SUV39H2 and MLL-WARD), DNA Methyltransferases (DNMT3A and DNMT3B) and Histone Demethylase (JMJD2A, JMJD2B, JMJD2C, JMJD2D, JMJD2E, JMJD3, JMJD1A, LSD1, Jarid1A, Jarid1B and Jarid1C) was performed by BPS Bioscience (http://www.bpsbioscience.com/index.ph). Binding experiments were performed in duplicate at each concentration.

HDAC1 and HDAC6 enzyme activity assays

HDAC1 and HDAC6 enzyme activities were measured with a specific fluorescence-labeled substrate (BPS Biosciences, Cat # 50037) after its deacetylation by HDACs. The fluorogenic substrate, containing an acetylated lysine side chain, can be deacetylated and then sensitized to subsequent treatment with the lysine developer, which produces a fluorophore that can be measured with a fluorescence plate reader. Human HDAC1 (GenBank Accession No. NM_004964), full length, with C-terminal His-tag and C-terminal Flag-tag, was obtained from BPS Biosciences (Cat. # 50051). Human HDAC6 (GenBank Accession number No. BC069243), full length with N-terminal GST tag was obtained from BPS Biosciences (Cat. # 50006). 5 µL of vehicle or tested compound 10 × concentrated prepared in assay buffer (BPS Biosciences, Cat # 50031) were added in black 96 well plates (final volume of 100 µL). The final percentage of DMSO was 1%. 5 µL

of HDAC1 (4 µg/mL) or HDAC6 (36 µg/mL) enzyme in assay buffer was added (final HDAC1 and HDAC6 concentration of 0.4 µg/mL and 3.6 µg/mL respectively) and the reaction was started by the addition of 40 µL of reaction mixture containing 0.125 mg/mL BSA (final concentration of 0.1 mg/mL) and 12.5 µM of fluorogenic HDACs substrate (final concentration of 10 µM). The reaction was incubated for 30 min at 37 ºC. After incubation, the reaction was stopped with 50 µL of lysine assay developer (BPS Biosciences, Cat # 50030). After incubation during 20 minutes at room temperature, the fluorescence of each well was measured at 355 nm excitation and 460 nm emission in a Mithras plate reader (Berthold). Positive control was obtained in the presence of the vehicle of the compounds. Negative control was obtained in the absence of HDAC enzyme activity. A best fit curve was fitted using GraphPad Prism 5 to derive the half maximal inhibitory concentration (IC_{50}) from this curve. Results are the average of two replicates.

CYP Inhibition

The inhibitory effect of CM-272 and CM-579 on five human cytochrome P450s (1A2, 2C9, 2C19, 2D6 and 3A4) was evaluated in human liver microsomes at Wuxi (http://www.wuxi.com/). Test compounds (10 µM) and the corresponding substrates for each P450 isoform (20 µL) were incubated with 140 µL liver microsomes (0.286 mg/mL; BD Gentest) and NADPH cofactor (20 µL, 1 mM) for 10 min at 37 ºC. The reaction was terminated by adding 400 µL cold stop solution (200 ng/mL tolbutamide in ACN) and samples were centrifuged at 4000 rpm for 20 minutes. Supernatants were analyzed by LC-MS/MS (Shimadzu LC 20-AD - API 4000), using peak area ratio of analyte/internal standard. Test compounds and positive controls were tested in duplicates. The percentage of inhibition was calculated as the ratio of substrate metabolite detected in treated and non-treated wells.

Plasma Protein Binding

This study was conducted by Equilibrium Dialysis at Wuxi (http://www.wuxi.com/). HT-Dialysis plate (Model HTD 96 b, Cat# 1006) and the dialysis membrane (molecular weight cut off 12-14 kDa, Cat# 1101) were purchased from HT Dialysis LLC (Gales Ferry, CT). Human plasma and CD-1 mouse plasma from Bioreclamation were thawed prior to experiment and the pH was adjusted at 7.4. Concentrations of test compounds in starting solution (before dialysis), plasma side of the membrane and buffer side of the membrane were quantified by LC-MS/MS (Shimadzu LC 20-AD - API 4000) methodologies, using peak area ratio of analyte/internal standard. The fraction of unbound, bound and recovery were calculated. Results are the average of three replicates.

Kinetic Solubility

This study was conducted at Wuxi (http://www.wuxi.com/) at pH 7.4 (50 mM phosphate buffer).

PAMPA Permeability

This study was conducted at Wuxi (http://www.wuxi.com/). PBS solution (100 mM phosphate, pH = 7.4) was prepared by dissolving 2.6 g of monobasic potassium phosphate and 14.1 g dibasic potassium phosphate in 1000 mL of ultra pure water. The pH was adjusted to 7.40±0.05 using either 1 M sodium hydroxide or 1 M hydrochloric acid. Test compounds were diluted to 0.2 mM in DMSO and further diluted prior to testing to 10 µM in PBS, pH 7.4 (final 5% DMSO). The assay was performed using a 96-well multiscreen-IP PAMPA plate (Millipore, Cat. # MAIPN4510), whose PVDF membrane was pre-coated with 5 µL of 1% lecithin/dodecane mixture. 150 µL of PBS solution containing the test compounds were added to the donor chamber. Blank PBS (300 µL) was also added to the PTFE acceptor plate, and the donor and acceptor plates were assembled and incubated for 4 h at room temperature with shaking at 150 rpm. Concentrations of test compound in starting solution, donor solution and receiver solution were quantified

by LC-MS/MS methodologies (Waters Aquity UPLC, - API4000) using peak area ratio of analyte/internal standard. Experiments were performed in duplicate.

Human and Mouse Liver Microsomal Stability

The data collected are analyzed to calculate a half-life ($t_{1/2}$, min) for test compounds at a final concentration of 1 μM. 5 μL of stock solution of test compound (10 mM) were diluted in 495 μL of 1:1 Methanol/Water (final concentration of 100 μM, 50% MeOH). Then, 50 μL of this intermediate solution were diluted in 450 μL of 100 mM potassium phosphate buffer to a concentration of 10 μM (working solution, 5% MeOH). The NADPH regenerating system contains β-Nicotinamide adenine dinucleotide phosphate (Sigma, Cat.# N0505), Isocitric acid (Sigma, Cat.#I1252) and Isocitric dehydrogenase (Sigma, Cat.#I2002) at a final concentration of 1unit/mL at incubation. Human liver microsomes were obtained from BD Gentest (Cat.#452117) and mouse liver microsomes from Xenotech (Cat.#M1000), to a final concentration of 0.7 mg protein /mL. A volume of 10 μL of working solution and 80 μL of microsome solution were added to a 96-well plate and incubated for 10 min at 37ºC. The reaction was started by the addition of 10 μL of NADPH regenerating system and stopped by the addition of 300 μL of stop solution (ACN at 4 ºC, including 100 ng/mL Tolbutamide and 100 ng/mL of Labetalol as internal standard) at different incubation times (0, 5, 10, 20, 30 and 60 min). Concentrations of test compound were quantified by LC-MS/MS methodologies (Shimadzu LC 20-AD / API4000) using peak area ratio of analyte/internal standard and the percent loss of parent compound was calculated under each time point to determine the half-life. Experiments were performed in duplicate.

hERG blockade assay

The effect of the compound on hERG potassium channels was determined using the PredictorTM hERG fluorescence polarization commercial assay kit (Life technologies cat#PV5365). The assay was carried out in black 384-well plates (Corning cat#3677 PS), monitoring changes of fluorescence polarization properties of the labeled hERG ligand between its soluble and bound states. The compound, which will compete with the fluorescent labeled hERG, was solubilized in 100% DMSO at a 16-points concentration curve way and then diluted 1/25 with hERG assay buffer. The assay contains 5 µL/well of studied compound, 10 µL/well of hERG membranes and 5 µL/well of hERG Tracer Red. Plate was incubated 2 h at room temperature, protected from light. Fluorescence polarization signals were recorded with an Envision plate reader (Perkin-Elmer). Experiment was performed once.

Cytotoxicity in THLE-2 cells

Cytotoxic effects of assayed compounds were tested using the immortalized human liver cell line THLE-2 (ATCC CRL-2706). Cells were cultured in BEGM medium (Clonetics #CC-4175) containing all the supplements kit except additional EGF and G418. Medium was completed by adding 0.7 µg/mL phosphoethanolamine, 0.5 ng/mL epidermal growth factor, antibiotics (penicillin and streptomycin) and 10% fetal bovine serum (FBS).

Cells were plated in 96-well black microplates at 10,000 cells/well density and were incubated at 37 ºC (5% CO2, 95% humidity) for 24 h to allow the cells to adhere and form a monolayer. Test compounds were solubilized in 100% DMSO at a concentration curve way and then diluted with cell culture medium containing 10% DMSO. The final concentrations of the test compounds (1% DMSO) ranged from 0-100 µM in a final volume of 200 µL. Microplates were maintained at 37 ºC (5% CO2, 95% humidity) during 3 days. Following this 72 h exposure to test compounds, cell viability in each well was determined by measuring the concentration of cellular adenosine triphosphate (ATP) using the ATP1Step Kit as described by the manufacturer (Perkin-Elmer). In a typical procedure, 50 µL of cell reagent is added to all wells of

each test plate followed by incubation for 10 min at room temperature on an orbital shaker. ATP concentration was determined by reading chemical luminescence using the Envision plate reader (PerkinElmer). The percentage of viable cells relative to the non-drug treated controls was determined for each well and LC_{50} values were calculated as concentrations projected to kill 50% of the cells following a 72 h exposure. Results are the average of two independent replicates (pLC_{50} difference < 0.3 log units). Cell lines were tested for mycoplasma (MycoAlert Sample Kit, Cambrex), no positive results were obtained.

Cytotoxicity in PBMCs

Cytotoxic effects of assayed compounds were tested using peripheral blood mononuclear cells (PBMCs) isolated following the regular density gradient centrifugation procedure with Ficol.

Cells were plated in 96-well black microplates at 100,000 cells/well density with DMEM medium (containing 10% FBS and antibiotics) and were incubated at 37 ºC (5% CO2, 95% humidity) for 24 h. Test compounds were solubilized in 100% DMSO at a concentration curve way and then diluted with cell culture medium containing 10% DMSO. The final concentrations of the test compounds (1% DMSO) ranged from 0-100 µM in a final volume of 200 µL. Microplates were maintained at 37 ºC (5% CO2, 95% humidity) during 3 days. Following this 72 h exposure to test compounds, cell viability in each well was determined by measuring the concentration of cellular adenosine triphosphate (ATP) using the ATP1Step Kit as described by the manufacturer (Perkin-Elmer). In a typical procedure, 80 µL of cell reagent is added to all wells of each test plate followed by incubation for 10 min at room temperature on an orbital shaker. ATP concentration was determined by reading chemical luminescence using the Envision plate reader (PerkinElmer). The percentage of viable cells relative to the non-drug treated controls was determined for each well and LC_{50} values were calculated as concentrations projected to kill 50% of the cells following a 72 h exposure. Results are the average of two independent replicates (pLC_{50} difference < 0.3 log units). Cell lines were tested for mycoplasma (MycoAlert Sample Kit, Cambrex), no positive results were obtained.

Pharmacokinetic study of CM-272 and CM-579 in plasma samples

CM-272 and CM-579 were measured in plasma samples using a Xevo-TQ MS triple quadropole mass spectrometer with an electrospray ionization (ESI) source and an Acquity UPLC (Waters, Manchester, UK).

CM-272 and CM-579 solutions were prepared by dissolving the solid in saline. A drug dosage of 1 mg/kg and 2.5 mg/kg (CM-272) or 1 mg/kg (CM-579) was administered as a single intravenous injection. Blood was collected at predetermined times over 24 h post injection (0.25, 2, 4, 6, 8 and 24 h for CM-272) and (0.25, 1, 2, 4 and 8 h for CM-579) into tubes containing EDTA and plasma was obtained via centrifugation (4 ºC, 2500 rpm, 5 min) and stored at -80 ºC until analysis.

Chromatographic separation was performed by gradient elution at 0.6 mL/min using an Acquity UPLC BEH C18 column (50 x 2.1 mm, 1.7 µm particle size; Waters). The mobile phase consisted of A: water with 0.1% formic acid, B: methanol with 0.1% (CM-272) or 0.08% (CM-579) formic acid. The auto-sampler temperature was set at 7 ºC and column temperature at 50 ºC. For detection and quantification, the electrospray ionization operated in the positive mode, and the collision gas used was ultra-pure argon at a flow rate of 0.15 mL min−1. The compound was detected using multiple reactions monitoring (MRM).

Quantification was achieved by external calibration using matrix-matched standards. Concentrations were calculated using a weighted least-squares linear regression (W = 1/x). Calibration standards were prepared by adding the appropriate volume of diluted solutions of the compound (made in a mixture of methanol and water, 50:50, v:v) to aliquots of 25 µL of blank plasma. 2% formic acid in acetonitrile was added to

precipitate the proteins. The mixture was then agitated for 5 min and centrifuged at 13200 rpm for 10 min at 4 ºC. A 5 μL aliquot of the resulting supernatant was injected onto the LC-MS/MS system for analysis. Frozen plasma samples were thawed at room temperature, vortexed thoroughly and subjected to the above described extraction procedure.

The pharmacokinetic parameters were obtained by fitting the blood concentration-time data to a non-compartmental model with the WinNonlin software (Pharsight, Mountain View, CA). For CM-272, plasma concentration-time profile for a dosing regimen of 2.5 mg/kg administered intravenously every 24 h for 3 weeks was simulated with Winnonlin.

Kinase Selectivity Profiling

The selectivity profiling of CM-272 against a selected panel of 97 kinases distributed through the kinome (out of which 90 are non-mutant kinases) was performed at DiscoverRx (http://www.discoverx.com/home) using the KINOME*scan*™ screening platform at a test concentration of 10 μM. Results for primary screen binding interactions are reported as 'Percent Control', calculated as *Percent Control* = (CM-272 signal – positive control signal) / (negative control signal – positive control signal) *100, where negative control corresponds to DMSO treatment (100%) and positive control to the signal obtained with a control competitive compound (0%).

For those targets with a Percent Control < 1%, the highest probability to obtain the most potent binding constants (Kd) *vs* assayed compound (CM-272), Kds were calculated with a standard dose-response curve using the Hill equation. Each target was assayed in duplicate.

Direct Binding Analysis

MicroScale Thermophoresis (MST) was performed to quantify biomolecular interactions between CM-579 and DNMT1 (full length). This is an immobilization-free assay, close-to-native conditions; in fact, identical to the biochemical assay (buffer described above). DNMT1 was labeled with Labeling Kits provided by NanoTemper Technologies, where the fluorescent red dye NT-647 was coupled via NHS coupling, and its concentration was kept constant (20nM). For performing this experiment, we have kept the concentration of NT-647 labeled DNMT1 constant, while the concentration of the non-labeled CM-579 was varied between 10 µM – 0.3 nM. After a short incubation the samples were loaded into MST NT.115 standard glass capillaries and the MST analysis was performed using the Monolith NT.115 instrument (NanoTemper Technologies). Results are the average of three independent measurements.

Cell culture

Almost all cell lines were maintained in culture in RPMI 1640 medium supplemented with fetal bovine serum at 37 °C in a humid atmosphere containing 5% CO_2. OCI-AML-2, J82, MGH-U3, UM-UC-1, MCF-7, U87-MG, 451-LU, 1205-LU, A375, SK-MEL-19, SK-MEL-28, SK-MEL-103, SK-MEL-147, WM-35, A498 and CAKI-2 were cultured with DMEM medium. OCI-Ly7 and OCI-Ly1 were maintained in culture in IMDM medium, JJN3 in IMDM + DMEM medium and OCI-Ly10 and OCI-Ly3 in IMDM supplemented with 20% human serum and 55 μM of β-mercaptoethanol. Cell lines were obtained from the DSMZ or the American Type Culture Collection (ATCC). All cell lines were authenticated by performing an STR (short tandem repeat) allele profile and were tested for mycoplasma (MycoAlert Sample Kit, Cambrex), no positive results were obtained.

Cell proliferation assay

Cell proliferation was analyzed using the CellTiter 96 Aqueous One Solution Cell Proliferation Assay (Promega, Madison, W). This is a colorimetric method for determining the number of viable cells in

proliferation. For the assay, suspension cells were cultured by triplicate at a density of $1x10^6$ cells/mL in 96-well plates (100.000 cells/well, 100µL/well), except for OCI-Ly3 and OCI-Ly10 cell lines which were cultured at a density of $0.5x10^6$ cells/mL (50.000 cells/well, 100µL/well). Adherent cells were obtained from 80-90% confluent flasks and 100 µL of cells were seeded at a density of 5000 cells /well in 96-well plates by triplicate. Before addition of the compounds, adherent cells were allowed to attach to the bottom of the wells for 12 hours. In all cases, only the 60 inner wells were used to avoid any border effects.

After 12, 24, 48 or 72 hours of treatment, plates with suspension cells were centrifuged at 800 g for 10 minutes and medium was removed. The plates with adherent cells were flicked to remove medium. Then, cells were incubated with 100 µL/well of medium and 20 µL/well of CellTiter 96 Aqueous One Solution reagent. After 1-3 hours of incubation at 37 ºC, the plates were incubated for 1-4 hours, depending on the cell line at 37 ºC in a humidified, 5 % CO_2 atmosphere. The absorbance was recorded at 490 nm using 96-well plate readers until absorbance of control cells without treatment was around 0.8. The background absorbance was measured in wells with only cell line medium and solution reagent. First, the average of the absorbance from the control wells was subtracted from all other absorbance values. Data were calculated as the percentage of total absorbance of treated cells/absorbance of non-treated cells. The GI_{50} values of the different compounds were determined using non-linear regression plots with the GraphPad Prism v5 software.

Cell cycle analysis

For cell cycle analysis, 250.000 cells were cultured at a density of $1x10^6$ cells/mL and treated for 12 and 24 hours with CM-272 at different concentrations (GI_{25}, GI_{50} and GI_{75}). Then, cells were washed twice with phosphate-buffered saline (PBS) and resuspended in 0.2 % Tween-20 in PBS and 0.5 mg/mL Rnase A (Ribonuclease A Type III-A from bovine pancreas, Cat No R5125, Sigma) and incubated for 30 min at 37 °C. Subsequently, cells were stained with 25 µg/mL of propidium iodide (Cat No P4170, Sigma) and analyzed using a BD FACSCalibur flow cytometer (Becton Dickinson, San Jose, CA, USA).

Apoptosis assay

For apoptosis assay, 100.000 cells of CEMO-1, LAL-CUN-2, MV4-11 and OCI-AML-2 cell lines were cultured at a density of $1x10^6$ cells/mL and treated for 12, 24, 48 and 72 hours with CM-272 at different concentrations (GI_{25}, GI_{50} and GI_{75}). In the case of OCI-Ly3 and OCI-Ly10, cells were treated with 100, 400 and 1000 nM for 24, 48, 72 and 96h. CEMO-1 cells were also treated with UNC0638, BIX-01294, 5-azacytidine or decitabine for 48 h at its GI_{50} (4.7 µM, 2.9 µM, 7.7 µM and 490 nM, respectively), 250 nM or 10 µM; MV4-11 cell line was treated with 270 nM and 10 µM of UNC0638, BIX-01294, 5-azacytidine or decitabine for 48 h; OCI-Ly10 cells were treated with 400 nM and 1 µM of UNC0638, BIX-01294, 5-azacytidine or decitabine for 48 h.

The FITC Annexin V Apoptosis Detection Kit I (Cat. No. 556419, BD Pharmingen) was used following the manufacturer's instructions, with some modification. Firstly, cells were washed twice with phosphate-buffered saline (PBS) and resuspended in 1X Binding Buffer at a concentration of 1x106 cells/mL. 1 µL of FITC Annexin V (AV) antibody and 2 µL of propidium iodide (PI) were added and incubated for 15 min at RT in the dark. Finally, 400 µL of 1X Binding Buffer were added to each tube and analyzed by flow cytometry within 1 h. We represented the addition of FITC AV positive and PI negative cells (early apoptosis) and FITC AV positive and PI positive cells (end stage apoptosis, death).

Combination assays

For the calculation of combination index (CI) values, OCI-AML-2 growth inhibition was determined at multiple concentrations of G9a inhibitor (A-366) (12.5, 25, 50 and 100µM) in combination with varied concentrations of Decitabine (12.5, 25, 50 and 100µM). Briefly, 100,000 cells were cultured at a density of $1x10^6$ cells/mL in duplicate in a 96 well plate. Cells were added in 80µL per well and the different concentrations of compounds were added in 10µL, being the final volume of 100µL. We prepared serial

dilutions 10 times concentrated and 10µL of each dilution is added per well. After addition of compounds, cells were incubated for 48 hours and then processed for MTS assays using the Cell Titer 96 Aqueous One Solution Cell Proliferation Assay (Cat. No. G3580, Promega) following the manufacturer's instructions. The resulting data were analyzed according to the method described by Chou (Calcusyn software, Biosoft). CI was used to determine whether the effect of drug combinations were synergistic, additive or antagonistic. Synergy, additivity and antagonism were defined by a CI less than one, one and greater than one, respectively.

For interference studies using siRNAs against G9a and DNMT1 cells were passaged 24 hours before nucleofection so that cells were in their logarithmic growth phase. The transfection of siRNAs was done with the Nucleofector II device (Amaxa GmbH, Köln, Germany) following the Amaxa guidelines. Briefly, 1×10^6 of CEMO-1, OCI-AML-2 and MV4-11 cells were resuspended in 100 µL of supplemented culture medium with 50nM of G9a or DNMT1 siRNAs or Silencer Select Negative Control-1 siRNA (Ambion, Austin, TX) and nucleofected with the Amaxa nucleofector apparatus using programs G-009, A-033 and A-030, respectively. We used two different siRNAs against each target (siG9a 1: CGCUGAUUUUCGAGUGUAA; siG9a2: CCAUGAACAUCGAUCGCAA; siDNMT1 A: DNMT1 siRNA h , Cat. No. sc35204, SantaCruz Biotechnologies; siDNMT1 B: GGAAGAAGAGUUACUAUAA) to demonstrate that the results obtained with siRNA nucleofection are not due to a combination of inconsistent silencing and sequence specific off-target effects. Silencer Select Negative Control-1 siRNA was used to demonstrate that the nucleofection did not induce non-specific effects on gene expression. Nucleofection was performed twice with a 24 hours interval. Cell proliferation was analyzed 48 hours after two repetitive transfections. Transfection efficiency was determined by flow cytometry using the BLOCK IT Fluorescent Oligo (Invitrogen Life Technologies, Paisley, UK).

Western blot

After 48 hours of treatment, cells were washed twice with PBS, being the last centrifugation of 4000 rpm for 10 min at 4 ºC. Histone extraction was performed as recommended by Upstate Biotechnology. Briefly, cells were homogenized in 5 volumes of lysis buffer (10 mM HEPES, pH 7.9; 1.5 mM $MgCl_2$; 10 mM KCl; 0.5 mM DTT; protease inhibitor cocktail (Complete Mini, Cat No 11836153301, Roche) and HCl was added to a final concentration of 0.2 M. After incubation on ice for 30 min, the homogenate was centrifuged at 11000 g for 10 min at 4 ºC, and the supernatant was first dialyzed twice against 0.1 M glacial acetic acid (1 hour each time) and then three times against water for 1 hour, 3 hours and o/n, respectively. The histone concentration in the extract was measured using the dye-binding assay of Bradford. 10 µg of histone was separated on 15 % SDS-PAGE gel and transferred to a nitrocellulose membrane. The membrane, after being blocked with Tropix I-block blocking reagent (Cat No AI300, Tropix) in PBS with 0.1 % of Tween-20 and 0.02 NaN_3, was incubated with the primary antibody against H3K9me2 (Mouse monoclonal antibody to Histone H3 dimethyl K9, Cat No ab1220, Abcam) diluted 1:2000 o/n at 4 ºC, against H3K27me3 (Mouse monoclonal antibody to Histone H3 trimethyl K27, Cat No ab6002, Abcam) diluted 1:2000 in BSA o/n at 4ºC, H3K36me3 (rabbit polyclonal antibody to Histone H3 trimethyl K36, Cat No ab9050, Abcam) diluted 1:2000 o/n at 4 ºC, H3K4me3 (rabbit polyclonal antibody to Histone H3 trimethyl K4, Cat No ab8580, Abcam) diluted 1:2000 o/n at 4 ºC, H3K79me3 (rabbit polyclonal antibody to Histone H3 trimethyl K79, Cat No ab2621, Abcam) diluted 1:2000 o/n at 4 ºC or Acetyl H3 (rabbit polyclonal antibody, Cat No 06-599, Millipore) diluted 1:50000 o/n at 4ºC and then with alkaline phosphatase-conjugated secondary antibodies. Bound antibodies were revealed by a chemiluminiscent reagent (Tropix) and detected using HyperfilmTM enhanced chemilumincescence. Total H3 was used as a loading control (diluted 1:50000 o/n at 4 ºC or for 1 hour at RT) (Anti-Histone H3, CT, pan, rabbit polyclonal, Cat No 07-690, Millipore). Images have been cropped for presentation. Full size images are presented in Supplementary Figure 13 and 14.

Dot blot

After 48 h of treatment, cells were washed twice with PBS and genomic DNA was extracted using a DNA kit (Nucleo Spin Tissue, Cat No 74095250, Macherey-Nagel) following the manufacturer's instructions.

DNA purity and concentration was measured using a NanoDrop spectrophotometer (Thermo Scientific). 500 ng of genomic DNA was loaded onto a nitrocellulose membrane (Amersham Hybond_N+, RPN203B, GE Healthcare), pre-wetted in 6X SSC for 10 min, using the Bio-Dot microfiltration apparatus (Cat No 170-6545, BioRad) following the manufacturer's instructions. Then the membrane was incubated with 2X SSC for 5 min and was cross-linked for 2 h at 80 ºC. The membrane, after being blocked with Tropix I-block blocking reagent (Cat No AI300, Tropix) in PBS with 0.1 % of Tween-20 and 0.02 NaN_3, was incubated with the primary antibody against 5-methylcytosine (Monoclonal antibody 5-Methylcytidine, Cat No BI-MECY-1000, Eurogentec) diluted 1:4000 o/n at 4 ºC and then with alkaline phosphatase-conjugated secondary antibody. Bound antibodies were revealed by a chemiluminiscent reagent (Tropix) and detected using HyperfilmTM enhanced chemilumincescence.

RNA-seq: alignment of Single-end Reads

The RNA of CEMO-1 and OCI-Ly10 cell lines before and after CM-272 treatment were sequenced by the standard Illumina protocol to create raw sequence files (.fastq files), which underwent quality control analysis using FastQC (http://www.bioinformatics.babraham.ac.uk/projects/fastqc/). To avoid low quality data negatively influencing downstream analysis, we trimmed the reads on the 3′-end and only used the first 51 bp from the 5′-end of each read for further analysis. We aligned the quality checked reads to the Human Feb. 2009 (GRCh37/hg19) genome using TopHat version 2.0.9 allowing for unique alignments to the genome and up to two mismatches. The resulting alignments were summarized by Ensembl gene identifiers to evaluate number of uniquely aligned reads per transcript and per sample (raw read counts).

RNA-seq data were analyzed using the limma package version 1.8.2 available through the Bioconductor open source. The raw read counts were used as input to form a DGEList object combining the counts and associated annotation. Scale normalization was applied and the calculation of normalized signal was performed by voom function of the limma package available through the Bioconductor open source software.

These data can be downloaded from Gene Expression Omnibus (GEO) public functional genomics data repository under the accession number GSE78932.

Expression arrays

The cells were harvested with TRIzol Reagent (Invitrogen) and the RNA was extracted according to the manufacturer's instructions. As a last step of the extraction procedure, the RNA was purified with the RNeasy Mini-kit (Qiagen, Hilden, Germany). Before cDNA synthesis, RNA integrity from each sample was confirmed on Agilent RNA Nano LabChips (Agilent Technologies).

The sense cDNA was prepared from 200 ng of total RNA and then fragmented and biotinylated using Affymetrix GeneChip® WT PLUS Reagent Kit. Labeled sense cDNA was hybridized to the Affymetrix Human Gene 2.0 ST microarray according to the manufacturer protocols and using GeneChip® Hybridization, Wash and Stain Kit. Genechips were scanned with the Affymetrix GeneChip® Scanner 3000.

Both background correction and normalization were done using RMA (Robust Multichip Average) algorithm. After quality assessment a filtering process was performed to eliminate low expression probe sets. Applying the criterion of an expression value greater than 16 in 2 samples for each experimental condition (NoTrt and CM272), 29737 probe sets in CEMO-1 cell line and 35305 probe sets in MV4-11 cell line were selected for statistical analysis. R and Bioconductor were used for preprocessing and statistical analysis.

LIMMA (Linear Models for Microarray Data) was used to find out the probe sets that showed significant differential expression between experimental conditions. Genes were selected as significant using a criteria of p-value<0.01 and logFC > 0.56 in CEMO-1 cell line and B>0 in MV4-11 cell line.

These data can be downloaded from Gene Expression Omnibus (GEO) public functional genomics data repository under the accession number GSE78517.

Functional analysis

Functional enrichment analysis of Gene Ontology (GO) categories was carried out using standard hypergeometric test. The biological knowledge extraction was complemented through the use of Ingenuity Pathway Analysis (Ingenuity Systems, www.ingenuity.com), which database includes manually curated and fully traceable data derived from literature sources.

IFN gene signature analysis

The fold change ranked list of genes was used as input to the non-parametric Kolmogorov-Smirnoff rank test as implemented in the GSEA (Gene Set Enrichment Analysis) software. GSEA allowed us to determine the enrichment of previously described IFN gene signatures.

Quantitative RT-PCR (Q-RT-PCR)

The expression of *IFI44L*, *EPSTI1*, *OASL*, *IFI6*, *USP18*, *ABTB2*, *PRDM5*, *NDNF* and *linc582* were analyzed by Q-RT-PCR in CEMO-1 and MV4-11 cell lines after 3, 6, 12, 24 and 48h of CM-272 treatment. First, cDNA was synthesized from 1 µg of total RNA using the PrimeScript RT reagent kit (Perfect Real Time) (Cat No RR037A, TaKaRa) following the manufacturer's instructions. The quality of cDNA was checked by a multiplex PCR that amplifies *PBGD*, *ABL*, *BCR* and *β2-MG* genes. Q-RT-PCR was performed in a 7300 Real-Time PCR System (Applied Biosystems), using 20 ng of cDNA in 2 µL, 1 µL of each primer at the concentration specified in Supplementary Table 15, 6 µL of SYBR Green PCR Master Mix 2X (Cat No 4334973, Applied Biosystems) in 12 µL reaction volume. The following program conditions were applied for Q-RT-PCR running: 50 ºC for 2 min, 95 ºC for 60 s following by 45 cycles at 95 °C for 15 s and 60 °C for 60 s; melting program, one cycle at 95 °C for 15 s, 40 °C for 60 s and 95 °C for 15 s. The relative expression of each gene was quantified by the Log $2^{(-\Delta\Delta Ct)}$ method using the gene *GUS* as an endogenous control.

Supplementary Table 15: Primers used for the Q-RT-PCR.

Gene	Primer Sequence	Stock concentration	Final concentration
IFI44L	F: tgttggcaaaagtgaagcaa<br>R: tgaagaacctcactgcaatcat	10 µM	0.83 µM
USP18	F: cagcaacatgaagagagagca<br>R: ttaaggcagcaggtctgtcc	10 µM	0.83 µM
EPSTI1	F: aagcatttagagagcatcagca<br>R: gccacaaacagcactttgac	10 µM	0.83 µM
OASL	F: gactgcggagcccatcac<br>R: tcaggctcacatagacctcag	5 µM	0.42 µM
ABTB2	F: ggagatggtcatcaacaacg<br>R: ccgttgtaggggctgaagta	5 µM	0.42 µM

IFI6	F: agctggtctgcgatcctg<br>R: tacctatgacgacgctgctg	20 µM	1.67 µM
PRDM5	F: ttcatgaggcaccatctcag<br>R: ccaggtagccaatcagaagc	5 µM	0.42 µM
NDNF	F: cctttggagtggaagctgag<br>R: aaccggatggggaactagac	10 µM	0.83 µM
limc582	F: caccttgggtcagatgtgtg<br>R: tcgaggtcaagcagaaaagaa	10 µM	0.83 µM
GUS	F: gaaaatatgtggttggagagctcatt<br>R: ccgagtgaagatcccctttta	15 µM	1.25 µM

Quantitative - Chromatin immunoprecipitation (Q-ChIP)

For ChIP analysis, 10 millions of CEMO-1 and MV4-11 cells were treated with CM-272 (250nM for CEMO-1 and 270nM for MV4-11) for 48 h. The ChIP assay was performed as previously described (*23*). Q-ChIP was performed in a 7300 Real-Time PCR System (Applied Biosystems), using 0.5 µL of each 2 µM primers indicated in Supplementary Table 16 (final concentration of 0.083µM), 6 µL of SYBR Green PCR Master Mix 2X (Cat No 4334973, Applied Biosystems) in 12 µL reaction volume. The amount of DNA varies among genes and is specified in Supplementary Table 16. The following program conditions were applied for Q-ChIP running: 50 ºC for 2 min, 95 ºC for 60 s following by 45 cycles at 95 °C for 15 s and 60 °C for 60 s; melting program, one cycle at 95 °C for 15 s, 40 °C for 60 s and 95 °C for 15 s. The percentage of H3K9me2 of each gene was quantified calculating [($2^{(-\Delta\Delta Ct)}$ CM-272 sample / $2^{(-\Delta\Delta Ct)}$ control)*100].

Supplementary Table 16: Primers used for the Q-ChIP.

Gene	Primer Sequence	DNA concentration
IFI44L	F: agtgtggactgagtgaaggag<br>R: tgttcaaagccaagcctgtc	5 ng
EPSTI1	F: acttctccggctttgtaggt<br>R: gtaatgactgctgtgtggct	5 ng
OASL	F: tgagaccagcctgaccaaaa<br>R: cctcagcctccctagtagct	20 ng
USP18	F: gaagacacccgatccaagaa<br>R: tgcgattttaaggtccttgc	20 ng
IFI6	F: gcatcaccatccaaaggctt<br>R: agaccagtgaagcctgtgtt	20 ng

ABTB2	F: tgcaggtgaaaccacaaatg<br>R: tttccaagtccgtctggttc	20 ng
PRDM5	F: ggcaataagctggctttcag<br>R: ctgtaggggagtctcgcttg	20ng
NDNF	F: gcctttatcccggacttagc<br>R: ctagcttttcgccctacacg	20ng
linc582	F: agtggtggcaaatggagtg<br>R: tcacttccctgtatggtcctg	20ng

DNA methylation analysis

DNA methylation status of *CDKN2A* and *POU4F2* promoters was analyzed by pyrosequencing techniques.

First, AML MV4-11 and OCI-AML-2 cell lines were treated daily for 7 consecutive days with 32.5nM and 33.8nM of CM-272, respectively. Cells were washed twice with PBS and genomic DNA was extracted using a DNA kit (Nucleo Spin Tissue, Cat No 74095250, Macherey-Nagel) following the manufacturer's instructions. DNA purity and concentration was measured using a NanoDrop spectrophotometer (Thermo Scientific). 1 µg of genomic DNA was treated and modified using the CpGenome DNA modification Kit (Cat No S7820, Chemicon International) following the manufacturer's instructions.

After bisulphite modification, "hot start" PCR (PyroMark PCR Kit, Cat No 978703, Qiagen) was performed with a denaturalization at 95°C for 15 minutes and for 10 cycles consisting of denaturation at 94°C for 30s, annealing at 50ºC for 45s, and extension at 72°C for 45s, followed by 45 cycles consisting of denaturation at 94°C for 30s, annealing at 57ºC for 45s, and extension at 72°C for 45s followed by a final 10 min extension. This PCR was performed using 2µl of modified DNA, 12.5µl of 2X Buffer, 0.5µl of 10µM of each specific primer (final concentration of 0.2µM) (CDKN2A-F: 5'-GAGGGGTTGGTTGGTTATTAGA-3', CDKN2A-R: 5'-Biotin-TACAAACCCTCTACCCACCTAAAT-3', POU4F2-F: 5'- AGAGAAAAGGGGGGTAAGAGG-3' and POU4F2-R: 5'-Biotin-ACTCAAATAACCCTAATCCCAAATT-3') in a final volume of 25µl. The resulting biotinylated PCR products were immobilized to streptaviding Sepharose High Performance beads (GE healthcare) and processed to yield high quality ssDNA using the PyroMark Vacuum Prep Workstation (Biotage), according to the manufactureŕs instructions. The pyrosequencing reactions were performed using the PyromarkTM ID (Biotage) and sequence analysis was performed using the PyroQ-CpG analysis software (Biotage).

Determination of cell surface-exposed calreticulin by immunofluorescence

Cells were incubated for 48 h with or without CM-272 compound (125 and 250 nM for CEMO-1, 135 and 270 nM for MV4-11 cell line and 200 and 400 nM for OCI-Ly10). Then, cells were harvested and washed with ice-cold PBS, then incubated with a CRT-specific antibody (Abcam) diluted in cold blocking buffer (5 % BSA in PBS) for 30 min on ice, washed, and incubated with an FITC-conjugated antibody (Sigma) in blocking buffer for 30 min. Thereafter, cells were washed, stained with 1 µg/mL TO-PRO-3 (Life Technologies) in cold PBS for 5 min, and analyzed by means of a FACSCalibur cytofluorometer (BD Biosciences). First line statistical analyses were performed by using the CellQuest™ software (BD Biosciences), upon gating on TO-PRO-3 negative events characterized by normal forward and side scatter (living cells).

Determination of extracellular HMGB1 concentrations

Cells were incubated for 48 h with or without CM-272 compound (125 and 250 nM for CEMO-1, 135 and 270 nM for MV4-11 cell line and 200 and 400 nM for OCI-Ly10). Extracellular HMGB1 from 48 h cell culture supernatants was quantified by means of the HMGB1 ELISA Kit II (Shino Test Corp.), following the manufacturer's instructions.

***In vivo* experiments**

All animal studies had previous approval from the Animal Care and Ethics Committee of the University of Navarra, whereas experiments that used patient samples were approved by the Human Research Ethics Committees of University of Navarra.

The human ALL CEMO-1 xenograft mice model was generated by intravenously (i.v.) injection of $10x10^6$ cells diluted in 100 µL of saline solution in the tail vein of a 6-8-week-old female BALB/cA- $Rag2^{-/-}\gamma c^{-/-}$ mice (n=12). Three days after injection, antitumor treatment was started intravenously in 6 mice. 1 mg/kg or 2.5 mg/kg of CM-272 was injected daily for 28 consecutive days. The control group (n=6) received only saline solution (diluents of CM-272). The mice weight was controlled and stable during the treatment. Statistical results were calculated using the statistical software medcalc. Two complete *in vivo* replicates have been performed.

For the AML MV4-11 i.v. model, $10x10^6$ MV4-11 cells diluted in 100 µL of saline solution were injected in the tail vein of BALB/cA- $Rag2^{-/-}\gamma c^{-/-}$ mice between 6 and 8 weeks of age (n=16). 14 days after injection, treatment was started in 8 mice and 2.5 mg/kg of CM-272 was intravenously injected daily for 28 consecutive days. The control group (n=8) received only saline solution. The mice weight was controlled and stable during the treatment. Statistical results were calculated using the statistical software medcalc. Two complete *in vivo* replicates were performed.

The human DLBCL OCI-Ly10 xenograft mice model was generated by intravenously (i.v.) injection of $2.5x10^6$ human primary cells diluted in 100 µL of saline solution in the tail vein of a 6-8-week-old female BALB/cA- $Rag2^{-/-}\gamma c^{-/-}$ mice (n=12). One day after injection, antitumor treatment was started intravenously in 6 mice. 2.5 mg/kg of CM-272 was injected for 5 consecutive days followed by 2 resting days for 8 week. The control group (n=6) received only saline solution. The mice weight was controlled and stable during the treatment. Statistical results were calculated using the statistical software medcalc. Two complete *in vivo* replicates were performed.

CM-272 toxicity assay: hematological and liver parameters.

After treating Rag2-/-γc-/- mice with daily i.v. 2.5 mg/kg of CM-272 during 4 weeks, followed by a 7 days washout period, hematological and liver parameters were measured. Moreover, Rag2-/-γc-/- mice transplanted with CEMO-1 ALL cells. BALB/cA- Rag2-/-γc-/- mice between 6 and 8 weeks of age (n=5) were injected through the tail vein with $10x10^6$ CEMO-1 cells diluted in 100 µL of saline. After treatment with 2.5 mg/kg of CM-272 or saline (initiated at day 3) for 28 consecutive days followed by a 7 days washout period hematological and liver parameters were measured.

Hematological parameters, white blood cells (WBC), red blood cells (RBC), platelet count (PLT), hemoglobin and hematocrit, were measured on a Hemavet Hematology Analyzer (Drew Scientific).Liver enzymes (albumin (ALB), alkaline phosphatase (ALP), aspartate transaminase (AST), alanine transaminase (ALT)), cholesterol, bilirrubin, urea and bile acids levels were analyzed from serum mice using a C311 Cobas Analyzer (Roche Diagnostics).

Livers collected from mice were fixed in paraformaldehyde at 4% for 6-8h and washed twice with saline solution and stored in 70% ethanol. Samples were included in paraffin and 3µm serial sections were cut, deparaffinated and stained with hematoxylin-eosin.

Supplementary References

1. Chou TC. Theorical basis, experimental design, and computerized simulation of synergism and antagonism in drug combination studies. *Pharmacol Rev* **58**, 621-81 (2006).

2. Foulks JM, et al. Epigenetic drug discovery: targeting DNA methyltransferases. *J Biomol Screen* **17**, 2-17 (2012).

3. Chang Y, et al. Structural basis for G9a-like protein lysine methyltransferase inhibition by BIX-01294. *Nat Struct Mol Biol* **16**, 312-317 (2009)

4. Vedadi M, et al. A chemical probe selectively inhibits G9a and GLP methyltransferase activity in cells. *Nature chemical biology* **7**, 566-574 (2011)

5. Fabian MA, et al. A small molecule-kinase interaction map for clinical kinase inhibitors. *Nat* Biotechnol **23**, 329-336 (2005).
